# Standing and travelling waves in a spherical brain model: The Nunez model revisited

**DOI:** 10.1016/j.physd.2017.02.017

**Published:** 2017-06-15

**Authors:** S. Visser, R. Nicks, O. Faugeras, S. Coombes

**Affiliations:** a School of Mathematical Sciences, University of Nottingham, NG7 2RD, UK; b Wellcome Trust Centre for Biomedical Modelling and Analysis, RILD Building, University of Exeter, EX2 5DW, UK; c INRIA Sophia Antipolis Mediterannee, 2004 Route Des Lucioles, Sophia Antipolis, 06410, France

**Keywords:** Neuronal networks, Integral equations, Space dependent delays, Dynamic pattern formation on a sphere, Normal form computation, Symmetric bifurcation theory

## Abstract

The Nunez model for the generation of electroencephalogram (EEG) signals is naturally described as a neural field model on a sphere with space-dependent delays. For simplicity, dynamical realisations of this model either as a damped wave equation or an integro-differential equation, have typically been studied in idealised one dimensional or planar settings. Here we revisit the original Nunez model to specifically address the role of spherical topology on spatio-temporal pattern generation. We do this using a mixture of Turing instability analysis, symmetric bifurcation theory, centre manifold reduction and direct simulations with a bespoke numerical scheme. In particular we examine standing and travelling wave solutions using normal form computation of primary and secondary bifurcations from a steady state. Interestingly, we observe spatio-temporal patterns which have counterparts seen in the EEG patterns of both epileptic and schizophrenic brain conditions.

## Highlights

• New results for understanding the development of patterns in the Nunez model for EEG generation.
• New analysis of wave solutions in a delayed integro-differential equation posed on a sphere.
• Techniques utilised include symmetric bifurcation theory and centre manifold reduction.
• Bespoke numerical scheme used to probe the behaviour of standing and travelling waves.

## 1 Introduction

Modern neuroimaging methodologies give us a window on the activity of the brain that may reveal both structure and function. Despite the recent advances in technologies for magnetic resonance imaging (MRI) for assessing anatomy, and functional MRI for assessing functional changes over seconds or minutes, the historical predecessor of electroencephalography (EEG), and its more recent magnetic counterpart magnetoencephalography (MEG), is still a hugely practical non-invasive tool for studying brain activity on the millisecond time-scale. The compromise being the relatively poor centimetre scale spatial resolution. However, from a modelling perspective this can actually be beneficial as it favours a more coarse grained description of neural tissue without recourse to detailed neuronal modelling. Indeed this is the current mode of thinking in cognitive neuroscience studies where single scalp electrodes (used in arrays across the head) are used to record the activity of $∼10^{8}$ neurons. Models that capture the large scale dynamics of neural tissue are often referred to as neural field models, and see  [1] for a recent discussion.

The model is, in its most general setting, described as a dynamical system with space-dependent delays that invariably is thought of as an integro-differential equation—which reduces to a damped inhomogeneous wave equation for a particular choice of exponentially decaying spatial interactions. Perhaps, Paul Nunez was one of the first to realise the importance of modelling the long range cortico–cortico connections for generating the all important α-rhythm of EEG (an 8–13 Hz frequency)  [2]. Moreover, he recognised that because the cortical white matter system is topologically close to a sphere that a model that respected this (with periodic boundaries) should naturally produce standing waves (via interference)  [3], [4]. For a more recent perspective on this work see  [5].

Given the importance assigned by Nunez to the boundary conditions  [6], the model has, surprisingly, been studied more often than not in scenarios that have different topologies to that of the sphere, e.g.  [7], [8], [9], [10]. Understandably this facilitates both mathematical and numerical analyses, though the results have less relevance to the application of standing waves seen in EEG. One exception to this is the numerical study of Jirsa et al.  [11], though even here analysis and simulations are performed by using the less general partial differential equation (PDE) formulation of the model. Despite the significance of geometry in the Nunez model for understanding EEG, a thorough exploration of its pattern forming properties has not been performed since its inception roughly forty years ago. In this paper we undertake a first step along this path.

### 1.1 Neural fields and symmetry

Analysis of spatio-temporal patterns in dynamical systems goes hand in hand with identifying the various symmetries in the model. Both the internal structure of the model, lattice-structure for example, and the domain under consideration, e.g. a disk, will impact the system’s symmetries. Since neural fields are primarily studied on either infinite or periodic domains, the group of translations and rotations (Euclidean group) arises naturally in this setting. Ermentrout and Cowan used this to good effect in developing their original model for visual hallucinations in primary visual cortex (V1), arising from a Turing instability  [12]. Apart from describing the time-evolution of activity in a strictly anatomical space, neural fields have been extended to ‘feature spaces’, which allow one to represent abstract attributes of neural activity. An outstanding example of this are the models of V1 of Cowan and collaborators, who included the cortical columns’ orientation preference for visual input in the framework of neural fields. A detailed analysis of the shift-twist symmetry group, which is at the heart of these models, has yielded an understanding on the origin of visual hallucinations characterised as lattices of locally oriented contours or edges  [13], [14]. Extending the model to account for spatial frequency preference of the visual stimuli, a feature distinct from the orientation, has also resulted in the formulation of a neural field on a sphere  [15]. Yet the differences with Nunez’ interpretation are marked: Nunez focuses on direct anatomical connectivity rather than interactions in an abstract feature space. In this work, we follow Nunez and take the sphere as the physical domain of the neural field.

Spherical symmetries have a long history of application, e.g. they play a role in morphogenesis (how an initial spherical ball of cells develops into a mature shape)  [16], as well many other biological and physical systems including the understanding of tumour growth  [17], sphere buckling under pressure  [18], and Rayleigh–Bénard convection in a spherical shell  [19] to give but a few examples. Typical models for these systems take the form of PDEs, such as reaction–diffusion or Swift–Hohenberg, and the techniques for understanding bifurcations from spherically symmetric states have included group theory  [20], scientific computation  [21] and Turing instability analysis  [22]. For a further discussion we refer the reader to the article by Matthews  [23].

### 1.2 Role of time delays

In contrast to other studies of pattern formation on a sphere we are concerned not with PDEs, but rather with non-local models. The Nunez model can be parsimoniously expressed as an integro-differential equation with time delays. Although the very first formulations of neural fields already include transmission delays  [24], [25], they have often been disregarded in rigorous analysis, due to lack of a proper mathematical setting for these problems. Recently, Faugeras and collaborators made their first steps in formulating a rigorous framework for these models  [26], [27], [28]. Subsequently, van Gils et al. proved that this class of dynamical systems can be cast as abstract delay differential equations  [29]. As a result of this, many of the mathematical techniques developed for the analysis of ODEs and PDEs, such as Turing analysis, symmetric bifurcation theory, and centre manifold reduction can be adapted for use in the delayed integro-differential equation we study in this article.

In  [30] a delayed neural field is studied on a one-dimensional interval and symmetries are used to simplify the computations of spectral values and normal form coefficients for a pitchfork–Hopf bifurcation. The inhomogeneities in their model (i.e. the boundaries) complicate the analytical computation of eigenfunctions and critical normal form coefficients—numerics have to be used instead. As a contrast, we will employ a Turing analysis in this paper, which enables us to express the eigenfunctions in closed form using spherical harmonics. Consequently, we are able to identify closed expressions for the critical normal form coefficients, where numerics are only required for computing the eigenvalues as a solution of a transcendental equation (which is common practice for delayed systems).

Although we are able to perform the centre manifold reduction with minimal numerical effort, forward-time simulations of the model are a whole different challenge. Indeed, the toolbox of numerical schemes is as yet relatively underdeveloped and so here we apply a novel scheme for the simulation of (discretised) integro-differential equations on large meshes  [31]: linear features of Cubic-Hermite spline interpolation and numerical integration are exploited to express the majority of operations in sparse matrix–vector products.

### 1.3 Outline

In Section  2 we give a brief review of the relevant neocortical anatomy and physiology to set the scene for the mathematical formulation of the large-scale Nunez model of EEG. We consider the case that the anatomical connectivity function is invariant with respect to the symmetry group of the sphere and show this can naturally be constructed using a spherical harmonic basis set. The non-instantaneous interaction between cortical regions is described with the use of a space-dependent delay determined by the speed of an action potential along an axonal fibre. Next in Section  3 we perform a linear Turing analysis of the steady state to show that both spatial and spatio-temporal neural activity patterns can occur (as linear combinations of spherical harmonics), depending on the precise shape of the connectivity function and the speed of the action potential. The techniques from equivariant bifurcation theory, which enable us to identify the possible *planforms* that can arise at the bifurcation, are reviewed in Section  4. Similarly, in Section  5 we offer a comprehensive overview of the framework of sun–star calculus that is required to perform the centre manifold reduction and critical normal form computation in neural fields with time delays. We apply these techniques to our model in Section  6 to obtain explicit expressions for the critical normal form coefficients, which enable us to classify the system’s bifurcation. In particular, we determine the first Lyapunov coefficient of a Hopf bifurcation and, by continuation, we subsequently find two different codimension two bifurcations: a generalised Hopf bifurcation and a double Hopf bifurcation. Both bifurcations give rise to bistability in the system, which we investigate analytically as well as numerically. Finally in Section  7 we discuss natural extensions of the work in this paper.

## 2 A model of cortex with axonal delays

The columnar organisation of the neocortex has been appreciated for some time, and for a review see  [32]. These (internally connected) macrocolumns consist of $∼10^{6}$ neurons with similar response properties and tend to be vertically aligned into columnar arrangements of roughly 1–3 mm in diameter. Columns in cortical areas located far from one another, but with some common properties, may be linked by long-range, intracortical connections (1–15 cm). Thus, to a first approximation the cortex is often viewed as being built from a dense reciprocally interconnected network of corticocortical axonal pathways, of which there are typically 10^10^ in a human brain  [33]. These fibres make connections within the roughly 3 mm outer layer of the cerebrum, and this wrinkled and folded cortical structure contains about 10^10^ neurons. Approximately 80% of these connections are excitatory and the remainder inhibitory. Excitatory pyramidal cells generally send their myelinated axons to other parts of the cortex (forming the white matter), so that most long-range synaptic interactions are excitatory. Roughly 95% of these connections target the same cerebral hemisphere, whilst the remaining ones either cross the corpus callosum to the other hemisphere or connect to the thalamus. In contrast inhibitory interactions tend to be much more short-ranged. It is the combination of *local* synaptic activity and *non-local* interactions within the cortex that is believed to be the major source of large-scale EEG and MEG signals recorded at (or near) the scalp.

Perhaps the most definitive model of EEG generation to date is that of Paul Nunez (reviewed in  [4]), which has culminated in the brain-wave equation for EEG generation. Indeed this and more general neural field models (reviewed in  [1]) are the major frameworks for the forward generation of EEG signals. At heart these modern biophysical theories assert that EEG signals from a single scalp electrode arise from the coordinated activity of pyramidal cells in cortex  [34]. EEG resolution (from the scalp) is typically in the 6 cm range for unprocessed EEG and 2–3 cm for high resolution EEG  [35]. Thus the number of neurons contributing to each scalp electrode is expected to be roughly 10^9^ for unprocessed EEG and 10^8^ for high resolution EEG. These are arranged with their dendrites in parallel and perpendicular to the cortical surface. When synchronously activated by synapses at the distal dendrites extracellular current flows (parallel to the dendrites), with a net membrane current at the synapse. For excitatory (inhibitory) synapses this creates a sink (source) with a negative (positive) extracellular potential. Because there is no accumulation of charge in the tissue this distal synaptic current is compensated by other currents flowing in the medium causing a distributed source in the case of a sink and vice versa for a synapse that acts as a source. Hence, at the population level the potential field generated by a synchronously activated population of cortical pyramidal cells behaves like that of a dipole layer. The interneurons’ contribution to the electrical field, on the other hand, is negligible due to both the small cell volume and the lack of a clear dipolar (or other orientation-dominant) morphology.

Nunez has convincingly argued that the dynamics of neural membrane alone cannot credibly account for the robust human EEG rhythms seen in the 1–15 Hz range, primarily because there is no such thing as a fixed membrane time constant *in vivo* (since for voltage gated membrane ion channels this is a time and state dependent attribute). However, local delays arising from synaptic processing (seen in the rise and decay of post synaptic potentials) as well as global delays arising from action potential propagation along corticocortical fibres are believed to be far more important. The former typically have time-scales from 1 to 100 ms and the latter of up to 30 ms in humans. The Nunez model of EEG respects the physiology and anatomy described above and has been particularly successful for describing standing EEG waves. Indeed these motivate one form of the model as a damped inhomogeneous wave equation whereby standing waves arise naturally by interference in a system with periodic boundary conditions. Nunez considered each cortical hemisphere (together with its white matter connections) to be topologically equivalent to a sphere, with the speed of an action potential fixed for all fibres—thus ignoring known anisotropy in the form of a preferred anterior–posterior orientation  [3], [4]. The radius of each cortical hemisphere was calculated from the known surface area of $∼800\text{–}1500\;{\mathrm{cm}}^{2}$ as $R=\sqrt{A/\left(4\pi \right)}∼8\text{–}10\;\mathrm{cm}$. Taking a value for the corticocortical action potential speed in the range v∼6–9m/s Nunez used simple interference arguments (using an analogy with vibrations on a string) to predict that fundamental cortical frequencies (for standing waves) would lie in the range f∼13–25Hz, using the relationship $f=v/\left(\sqrt{2}\pi R\right)$. Another version of the model takes the form of an integro-differential equation and it is this formulation of the model that we shall consider in this paper.

### 2.1 The model

Here we give a modern perspective on the Nunez model in its integral form. Using some artistic licence we also slightly generalise it to include a simple model of synaptic processing, to bring it more in line with popular formulations of neural field theories. For a review of these we refer the reader to the recent book  [36].

We represent synaptic activity by u=u(t,r)∈R where r is a point on the surface of a sphere and $t\in R^{+}$. We shall consider simple neural field models that, after rescaling time and space, can be written in the form

(1) $$\frac{\partial u\left(t,r\right)}{\partial t}=-u\left(t,r\right)+\Phi \left(t,r\right)$$

with either

(2a) $$\Phi \left(t,r\right)=\int_{\Omega }\underline{w}\left(r,r^{\prime }\right)f\circ u\left(t-\underline{\tau }\left(r,r^{\prime }\right),r^{\prime }\right)dr^{\prime }\text{,}$$

or

(2b) $$\Phi \left(t,r\right)=f\circ \int_{\Omega }\underline{w}\left(r,r^{\prime }\right)u\left(t-\underline{\tau }\left(r,r^{\prime }\right),r^{\prime }\right)dr^{\prime }\text{.}$$

We also have to specify the initial condition:

(2c) u(t)=η(t),t∈[−h,0],

where η∈X, the state space defined below.

Here $\Omega ≔S^{2}$ is the surface of the unit sphere in $R^{3}$, $dr^{\prime }$ the integration measure and ∘ denotes function composition, with f a firing rate function. The weight distribution $\underline{w}\left(r,r^{\prime }\right)$ specifies the anatomical connectivity between points r and $r^{\prime }$, whilst $\underline{\tau }\left(r,r^{\prime }\right)$ specifies the corresponding delay arising from the finite speed of the action potential travelling along the fibre connecting the two points. The model defined by (2a) is often referred to as a voltage based model, whereas (2b) is referred to as an activity based model  [37]. In either case the models are qualitatively similar in their behaviour, and the analytical and numerical techniques we develop throughout this paper can be adapted to either case. For concreteness we shall work with (2a).

### 2.2 Functional analytic setting

Throughout this paper we fix the following assumptions:

• the *firing rate function* f is smooth and bounded on R,
• the *domain* $\Omega ≔S^{2}$ is the unit sphere in $R^{3}$ and the corresponding metric d is the great circle distance,
• the *connectivity kernel* $\underline{w}\in C^{0,\alpha }\left(\Omega \times \Omega \right)$, the Banach space of Hölder continuous functions with exponent α, 1/2<α≤1 on Ω×Ω.
• the *delay function* $\underline{\tau }\in C^{0,\alpha }\left(\Omega \times \Omega \right)$ is non-negative and not identically zero,
• the *maximal delay* $h≔\sup\left\{\underline{\tau }\left(r,r^{\prime }\right):r,r^{\prime }\in \Omega \right\}$,
• the *spatial space* $V≔C^{0,\alpha }\left(\Omega ,C\right)$ the Banach space of Hölder continuous functions with exponent α, 1/2<α≤1, with the standard norm:
  $${\left\|v\right\|}_{V}≔\underset{r\in \Omega }{\sup}\left|v\left(r\right)\right|+\underset{r\neq r^{\prime }\in \Omega }{\sup}\frac{\left|v\left(r\right)-v\left(r^{\prime }\right)\right|}{d{\left(r,r^{\prime }\right)}^{\alpha }}\text{,}$$
• the *state space* $X≔C^{0,1}\left(\left[-h,0\right],V\right)$ the Banach space of Lipschitz continuous functions with the standard norm:
  $${\left\|x\right\|}_{X}≔\underset{t\in \left[-h,0\right]}{\sup}{\left\|x\left(t,\cdot \right)\right\|}_{V}+\underset{t\neq t^{\prime }\in \left[-h,0\right]}{\sup}\frac{{\left\|x\left(t,\cdot \right)-x\left(t^{\prime },\cdot \right)\right\|}_{V}}{\left|t-t^{\prime }\right|}\text{,}\;\text{and}$$
• a function $u\in C\left(\left[-h,\infty \right),V\right)\cap C^{1}\left(\left[0,\infty \right),V\right)$ that satisfies (1) with initial condition η∈X is a *global solution*.

These assumptions are nearly identical to those in the seminal work  [29], apart from one: where the original work sets the spatial space V as the continuous functions, we have chosen the Hölder continuous functions instead. This additional constraint on the spatial domain is in our case required to guarantee the convergence of the spherical harmonics expansion (see next section). Moreover, we point out this change of spatial function space has no apparent impact on the outcomes of the original work and a discussion of these minute adjustments is therefore omitted.

We include for completeness the proofs of two lemmas that guarantee that the equations (1)  +  (2a)  +  (2c) are well-posed. Using the notations of  [29], define the nonlinear operator G:X→V as

(3) $$G\left(u\right)\left(r\right)=\int_{\Omega }\underline{w}\left(r,r^{\prime }\right)f\circ u\left(-\underline{\tau }\left(r,r^{\prime }\right),r^{\prime }\right)dr^{\prime }\text{.}$$

We have the following lemma.

Lemma 1 G:X→V *is well-defined by*   (3)*.*

Proof Given u∈X, we consider two points r and $\overset{¯}{r}$ of Ω and write

$$\left|G\left(u\right)\left(r\right)-G\left(u\right)\left(\overset{¯}{r}\right)\right|\leq \left|\int_{\Omega }\left(w\left(r,r^{\prime }\right)-w\left(\overset{¯}{r},r^{\prime }\right)\right)f\circ u\left(-\underline{\tau }\left(r,r^{\prime }\right),r^{\prime }\right)dr^{\prime }\right|+\left|\int_{\Omega }w\left(\overset{¯}{r},r^{\prime }\right)\left(f\circ u\left(-\underline{\tau }\left(r,r^{\prime }\right),r^{\prime }\right)-f\circ u\left(-\underline{\tau }\left(\overset{¯}{r},r^{\prime }\right),r^{\prime }\right)\right)dr^{\prime }\right|\overset{\text{Hölder continuity of}\;w}{\overset{\mathrm{Boundedness of}\;f}{\leq }}C_{f}L_{w}{\left\|r-\overset{¯}{r}\right\|}^{\alpha }+\left|\int_{\Omega }w\left(\overset{¯}{r},r^{\prime }\right)\left(f\circ u\left(-\underline{\tau }\left(r,r^{\prime }\right),r^{\prime }\right)-f\circ u\left(-\underline{\tau }\left(\overset{¯}{r},r^{\prime }\right),r^{\prime }\right)\right)dr^{\prime }\right|\overset{\mathrm{Boundedness of}\;w}{\overset{\mathrm{Smoothness of}\;f}{\leq }}C_{f}L_{w}{\left\|r-\overset{¯}{r}\right\|}^{\alpha }+C_{w}L_{f}\int_{\Omega }\left|u\left(-\underline{\tau }\left(r,r^{\prime }\right),r^{\prime }\right)-u\left(-\underline{\tau }\left(\overset{¯}{r},r^{\prime }\right),r^{\prime }\right)\right|dr^{\prime }\overset{u\;\mathrm{Lipschitz continuous in time}}{\overset{\underline{\tau }\;\text{Hölder}\;\mathrm{continuous}}{\leq }}C_{f}L_{w}{\left\|r-\overset{¯}{r}\right\|}^{\alpha }+C_{w}L_{f}L_{u}L_{\tau }{\left\|r-\overset{¯}{r}\right\|}^{\alpha }\leq C{\left\|r-\overset{¯}{r}\right\|}^{\alpha }\text{.}$$

 □

Using the definition of the operator G, the system (1)  +  (2a)  +  (2c) can be rewritten as the following initial value problem

(4) $$\left\{ \begin{array}{ccc} \overset{˙}{u}\left(t\right) & = & -u\left(t\right)+G\left(u_{t}\right)\;t\geq 0\\ u\left(t\right) & = & \Psi \left(t\right)\;t\in \left[-h,0\right]\text{.} \end{array}\right.$$

Then (4) is of the form of a Delayed Differential Equation when we define F:X→V by

(5) F(Ψ)=−Ψ(0)+G(Ψ)∀Ψ∈X.

It is then well-known that (4) has a unique solution or equivalently that (1)  +  (2a)  +  (2c) has a unique global solution if the operator F defined by (5) is Lipschitz continuous. We prove the following lemma.

Lemma 2 *The operator* F:X→V *defined by*(5)   *is Lipschitz continuous.*

Proof If Ψ and $\overset{¯}{\Psi }$ are elements of X and $r,\;\overset{¯}{r}\in \Omega$, consider

(6) $$\left|F\left(\Psi \left(r\right)\right)-F\left(\overset{¯}{\Psi }\left(r\right)\right)\right|\leq \left|\Psi \left(0,r\right)-\overset{¯}{\Psi }\left(0,r\right)\right|+\int_{\Omega }\left|w\left(r,r^{\prime }\right)\right|\;\left|f\left(\Psi \left(-\tau \left(r,r^{\prime }\right),r^{\prime }\right)\right)-f\left(\overset{¯}{\Psi }\left(-\tau \left(r,r^{\prime }\right),r^{\prime }\right)\right)\right|\;dr^{\prime }\overset{\mathrm{Boundedness of}\;w}{\overset{\mathrm{Smoothness of}f}{\leq }}\left|\Psi \left(0,r\right)-\overset{¯}{\Psi }\left(0,r\right)\right|+C_{w}L_{f}\int_{\Omega }\left|\Psi \left(-\tau \left(r,r^{\prime }\right),r^{\prime }\right)-\overset{¯}{\Psi }\left(-\tau \left(r,r^{\prime }\right),r^{\prime }\right)\right|\;dr^{\prime }\leq \left|\Psi \left(0,r\right)-\overset{¯}{\Psi }\left(0,r\right)\right|+C_{w}L_{f}\left|\Omega \right|\underset{t\in \left[-h,0\right]}{\sup}\underset{r\in \Omega }{\sup}\left|\Psi \left(t,r\right)-\overset{¯}{\Psi }\left(t,r\right)\right|\leq C_{1}{\left\|\Psi -\overset{¯}{\Psi }\right\|}_{X}\text{,}$$

for some positive constant $C_{1}$. Consider now

$$\frac{\left|F\left(\Psi \left(r\right)\right)-F\left(\overset{¯}{\Psi }\left(\overset{¯}{r}\right)\right)\right|}{d{\left(r,\overset{¯}{r}\right)}^{\alpha }}\overset{\mathrm{Boundedness of}\;w}{\overset{\mathrm{Smoothness of}f}{\leq }}\frac{\left|\Psi \left(0,r\right)-\overset{¯}{\Psi }\left(0,\overset{¯}{r}\right)\right|}{d{\left(r,\overset{¯}{r}\right)}^{\alpha }}+C_{w}L_{f}\int_{\Omega }\frac{\left|\Psi \left(-\tau \left(r,r^{\prime }\right),r^{\prime }\right)-\overset{¯}{\Psi }\left(-\tau \left(\overset{¯}{r},r^{\prime }\right),r^{\prime }\right)\right|}{d{\left(r,\overset{¯}{r}\right)}^{\alpha }}\;dr^{\prime }\text{.}$$

We also have

$$\left|\Psi \left(-\tau \left(r,r^{\prime }\right),r^{\prime }\right)-\overset{¯}{\Psi }\left(-\tau \left(\overset{¯}{r},r^{\prime }\right),r^{\prime }\right)\right|\leq {\left\|\Psi -\overset{¯}{\Psi }\right\|}_{X}\;\left|\tau \left(r,r^{\prime }\right)-\tau \left(\overset{¯}{r},r^{\prime }\right)\right|\overset{\tau \in C^{0,\alpha }\left(\Omega \times \Omega \right)}{\leq }{\left\|\Psi -\overset{¯}{\Psi }\right\|}_{X}\left\|\tau \right\|d{\left(r,\overset{¯}{r}\right)}^{\alpha }\text{,}$$

so that

(7) $$\frac{\left|F\left(\Psi \left(r\right)\right)-F\left(\overset{¯}{\Psi }\left(\overset{¯}{r}\right)\right)\right|}{d{\left(r,\overset{¯}{r}\right)}^{\alpha }}\leq C_{2}{\left\|\Psi -\overset{¯}{\Psi }\right\|}_{X}\text{,}$$

for some positive constant $C_{2}$. Combining (6), (7) we obtain

$${\left\|F\left(\Psi \right)-F\left(\overset{¯}{\Psi }\right)\right\|}_{V}\leq C{\left\|\Psi -\overset{¯}{\Psi }\right\|}_{X}\text{,}$$

for some positive constant C, i.e.  F is Lipschitz continuous. □

### 2.3 Spherical geometry

For the remainder of this paper we will assume that $r=r\left(\theta ,\phi \right)\in S^{2}$ is a point on the sphere with polar angle θ∈[0,π], azimuthal angle ϕ∈[0,2π) and radius 1 and similar for $r^{\prime }$. Furthermore, we make use of the complex-valued spherical harmonics $Y_{n}^{m}\left(r\right)$ of degree n≥0 and order |m|≤n, for which a representation is given in Appendix A. As much as Fourier series form an orthonormal basis on the circle, spherical harmonics form an orthonormal basis on the sphere.

Theorem 1 **Spherical Harmonics Expansion [38, Thm. 5]**

*Let* v∈V*, then the spherical harmonics expansion of* v *converges uniformly to* v*, that is for* N→∞

(8) $$\overset{N}{\underset{n=0}{\sum }}\overset{n}{\underset{m=-n}{\sum }}v_{n}^{m}Y_{n}^{m}\to v\;\text{uniformly on }\Omega \text{.}$$

*The coefficients* $v_{n}^{m}$ *are given by projections on the basis functions:*

(9) $$v_{n}^{m}≔\int_{\Omega }v\left(r\right)\bar{Y_{n}^{m}\left(r\right)}\;dr\text{,}$$

*where the overline denotes complex conjugation.*

Proof We give a very short proof for completeness. Let $P_{N}$ be the linear operator defined by $P_{N}\left(v\right)=\sum_{n=0}^{N}\sum_{m=-n}^{n}v_{n}^{m}Y_{n}^{m}$ and $E_{N}\left(v\right)$ be the infinite norm of the difference $v-P_{N}\left(v\right)$:

$$E_{N}\left(v\right)=\underset{r\in \Omega }{\sup}\left|\left(v-P_{N}\left(v\right)\right)\left(r\right)\right|\text{.}$$

Ref.  [39, Cor. 2.2] implies that $E_{N}\left(v\right)\leq C{\left\|v\right\|}_{V}N^{-\alpha }$, while Ref.  [38, Thm. 4] implies that the operator norm $\left\|P_{N}\right\|$ of the linear operator $P_{N}$ w.r.t. the uniform norm on V satisfies $\left\|P_{N}\right\|=O\left(N^{1/2}\right)$. Finally  [38, Thm. 1] shows that ${\left\|v-P_{N}\left(v\right)\right\|}_{\infty }\leq \left(1+\left\|P_{N}\right\|\right)E_{N}\left(v\right)$ and this yields

$${\left\|v-P_{N}\left(v\right)\right\|}_{\infty }=O\left(N^{-\alpha +1/2}\right)\text{,}$$

hence the uniform convergence on Ω of $P_{N}\left(v\right)$ to v when N→∞. □

We shall consider a homogeneous neural field, where both the weight kernel $\underline{w}\left(r,r^{\prime }\right)$ and transmission delays $\underline{\tau }\left(r,r^{\prime }\right)$ depend on the relative position of r and $r^{\prime }$. Naturally, w and τ are chosen as functions of distance along the surface, but more generally we set

(10) $$\underline{w}\left(r,r^{\prime }\right)≔w\left(r\cdot r^{\prime }\right)\;\text{and}\;\underline{\tau }\left(r,r^{\prime }\right)≔\tau \left(r\cdot r^{\prime }\right)\text{.}$$

We remark that, on a unit sphere, the inner product is equal to the cosine of the angular separation (and therefore great circle distance) between two points; i.e.  $\cos\left(\alpha \right)=r\cdot r^{\prime }$. This leads to the following theorem.

Theorem 2 *Let* g *be a Hölder continuous function on* [−1,1] *with exponent* α*,* 1/2<α≤1*, and* $G\left(r,r^{\prime }\right)=g\left(r\cdot r^{\prime }\right)$*. Then the series* ${\left(\sum_{n=0}^{N}G_{n}\sum_{m=-n}^{n}Y_{n}^{m}\left(r\right)\bar{Y_{n}^{m}\left(r^{\prime }\right)}\right)}_{N}$ *with coefficients*

(11) $$G_{n}≔2\pi \int_{-1}^{1}g\left(s\right)P_{n}\left(s\right)ds\text{,}$$

*where* $P_{n}$ *is the Legendre polynomial of degree* n*, converges to* $G\left(r,r^{\prime }\right)$*, uniformly on* Ω×Ω*, when* N→∞*.*

Proof It is easily checked that the hypothesis on the function g implies that G is Hölder continuous on Ω×Ω with exponent α:

$$\left|G\left(r_{1},r_{1}^{\prime }\right)-G\left(r_{2},r_{2}^{\prime }\right)\right|=\left|g\left(r_{1}\cdot r_{1}^{\prime }\right)-g\left(r_{2}\cdot r_{2}^{\prime }\right)\right|\overset{g\;\text{Hölder}\;\alpha }{\leq }k_{g}{\left|r_{1}\cdot r_{1}^{\prime }-\left(r_{2}\cdot r_{2}^{\prime }\right)\right|}^{\alpha }=k_{g}{\left|\left(r_{1}-r_{2}\right)\cdot r_{1}^{\prime }+\left(r_{1}^{\prime }-r_{2}^{\prime }\right)r_{2}\right|}^{\alpha }\leq k_{g}{\left(\left|\left(r_{1}-r_{2}\right)\cdot r_{1}^{\prime }\right|+\left|\left(r_{1}^{\prime }-r_{2}^{\prime }\right)r_{2}\right|\right)}^{\alpha }\overset{\text{Cauchy–Schwarz}}{\leq }k_{g}{\left(\left\|r_{1}-r_{2}\right\|+\left\|r_{1}^{\prime }-r_{2}^{\prime }\right\|\right)}^{\alpha }\overset{\mathrm{Jensen inequality}}{\leq }k_{g}\left({\left\|r_{1}-r_{2}\right\|}^{\alpha }+{\left\|r_{1}^{\prime }-r_{2}^{\prime }\right\|}^{\alpha }\right)\text{.}$$

An easy extension of Theorem 1 shows that the series ${\left(\sum_{n,n^{\prime }=0}^{N}\sum_{m,m^{\prime }=-n}^{n}G_{nn^{\prime }}^{mm^{\prime }}Y_{n}^{m}\left(r\right)\bar{Y_{n^{\prime }}^{m^{\prime }}\left(r^{\prime }\right)}\right)}_{N}$ converges uniformly to $G\left(r,r^{\prime }\right)$ when N→∞, where the coefficients $G_{nn^{\prime }}^{mm^{\prime }}$ are given by the projections:

(12) $$G_{nn^{\prime }}^{mm^{\prime }}=\int_{\Omega }\int_{\Omega }g\left(r\cdot r^{\prime }\right)Y_{n^{\prime }}^{m^{\prime }}\left(r^{\prime }\right)\bar{Y_{n}^{m}\left(r\right)}\;dr^{\prime }\;dr\text{.}$$

Since g is continuous on [−1,1] hence in $L_{1}$ (integrable), we can apply the Funk–Hecke theorem  [40, Vol. 2, p. 247] to (12) and obtain

(13) $$G_{nn^{\prime }}^{mm^{\prime }}=\int_{\Omega }\int_{-1}^{1}2\pi g\left(s\right)P_{n}\left(s\right)ds\;Y_{n^{\prime }}^{m^{\prime }}\left(r\right)\bar{Y_{n}^{m}\left(r\right)}\;dr$$

(14) $$=2\pi \;\delta_{nn^{\prime }}\delta_{mm^{\prime }}\int_{-1}^{1}g\left(s\right)P_{n}\left(s\right)\;ds\text{,}$$

due to orthogonality. □

We note that a similar mathematical representation has previously been used in  [14], [15] for a neural field model describing orientation and spatial frequency tuning in a cortical hypercolumn. However, the physical differences between our studies are marked, with ours focusing on direct anatomical connectivity rather than interactions in a neural feature space.

### 2.4 Concrete choices

While the majority of the following results are independent of the specific choices for f, w and τ, we will illustrate our results with concrete choices in computations and simulations. Where required, we choose the following forms.

The firing rate function is sigmoidal and increasing:

(15) $$f\left(u\right)=\frac{\alpha }{1+e^{-\beta \left(u-\delta \right)}}\text{,}$$

with steepness parameter β>0 and threshold δ.

A natural choice for the connectivity kernel is

(16) $$w\left(s\right)=J_{1}\exp\left(-\frac{\cos^{-1}s}{\sigma_{1}}\right)+J_{2}\exp\left(-\frac{\cos^{-1}s}{\sigma_{2}}\right)\text{,}$$

with $\sigma_{1}>\sigma_{2}>\sigma_{\min}>0$ and $J_{1}J_{2}<0$. For $J_{1}+J_{2}>0$ we have a wizard-hat shape, whilst for $J_{1}+J_{2}<0$ we have an inverted wizard-hat shape.

We have the following lemma.

Lemma 3 *The function* w:[−1,1]→R *defined by*   (16)   *is Hölder continuous with exponent* α*,* 1/2<α≤1*.*

Proof The space of Hölder continuous functions with the same exponent being a vector space, it is sufficient to check that $ww:s\to \exp\left(-\frac{\cos^{-1}s}{\sigma_{1}}\right)$ is Hölder continuous with exponent α. We have

$$\left|ww\left(s\right)-ww\left(s^{\prime }\right)\right|\leq \left|\exp\left(-\frac{\cos^{-1}s}{\sigma_{1}}\right)-\exp\left(-\frac{\cos^{-1}s^{\prime }}{\sigma_{1}}\right)\right|\text{.}$$

Since $-\frac{\pi }{2\sigma_{1}}\leq \frac{\cos^{-1}s}{\sigma_{1}}\leq \frac{\pi }{2\sigma_{1}}$, we have

$$\left|\exp\left(-\frac{\cos^{-1}s}{\sigma_{1}}\right)-\exp\left(-\frac{\cos^{-1}s^{\prime }}{\sigma_{1}}\right)\right|\leq \exp\left(\frac{\pi }{2\sigma_{1}}\right)\left|\frac{\cos^{-1}s}{\sigma_{1}}-\frac{\cos^{-1}s^{\prime }}{\sigma_{1}}\right|\leq \frac{1}{\sigma_{\min}}\exp\left(\frac{\pi }{2\sigma_{\min}}\right)\left|\cos^{-1}s-\cos^{-1}s^{\prime }\right|\text{.}$$

Since $s\to \cos^{-1}s$ is Lipschitz continuous, $\left|\cos^{-1}s-\cos^{-1}s^{\prime }\right|\leq C\left|s-s^{\prime }\right|=2C\left|\frac{s-s^{\prime }}{2}\right|$, for some positive constant C, and since $0\leq \left|\frac{s-s^{\prime }}{2}\right|\leq 1$ we have $\left|\frac{s-s^{\prime }}{2}\right|\leq {\left|\frac{s-s^{\prime }}{2}\right|}^{\alpha }$, 1/2<α≤1. It follows that

$$\left|ww\left(s\right)-ww\left(s^{\prime }\right)\right|\leq C{\left|s-s^{\prime }\right|}^{\alpha }\text{,}$$

for some positive constant C and for all $s,\;s^{\prime }\in \left[-1,1\right]$. □

Furthermore, we call the synaptic kernel *balanced* if

$$w_{0}=2\pi \int_{-1}^{1}w\left(s\right)ds=0\text{.}$$

Other choices than (16), such as a difference of Gaussians, are also natural.

A common choice for the transmission delay is

(17) $$\tau \left(s\right)=\tau_{0}+\frac{\cos^{-1}s}{c}\text{,}$$

which incorporates both a constant offset delay $\tau_{0}$ and a constant propagation speed c of action potentials. An onset delay has been shown to lead to dynamics reminiscent of those seen in simulations of large-scale spiking networks  [41], and its physiological interpretation can be connected to the relaxation time-scale for which spiking networks can reasonably allow for a firing rate description. We have the following lemma.

Lemma 4 *The function* τ *defined by*   (17)   *is Hölder continuous with exponent* α*,* 1/2<α≤1*.*

Proof It follows from the proof of Lemma 3 that τ is Hölder continuous with exponent α, 1/2<α≤1. □

## 3 Linear stability analysis

It is sensible to begin the investigation of the spherical Nunez model with a standard Turing analysis, treating the instability of a homogeneous steady state. This will allow us to determine the conditions for the onset of spatial patterned states or more dynamic spatio-temporal patterns in the form of standing or travelling waves. This approach has a long tradition in neural field modelling, as exemplified in  [12], [42], [13] and reviewed in  [37], [43]. A one-dimensional system with space-dependent delays and periodic domain is studied in  [28] using the same methodology.

### 3.1 Spectral problem

Stability and pattern formation in the model are dictated by the spectral values, or eigenvalues, of the semigroup generator underlying the dynamical system. We continue with a heuristic derivation of a set of characteristic equations $E_{n}\left(\lambda \right)$ whose roots are the eigenvalues of (1)  +  (2a). A detailed treatise on the validity of our result, as well as special properties of the spectral problem, is available in  [29].

A homogeneous steady state $u\left(t,r\right)=\overset{ˆ}{u}$ of (1)  +  (2a) satisfies

(18) $$\overset{ˆ}{u}=w_{0}f\left(\overset{ˆ}{u}\right)\text{.}$$

For our choice (15), up to three homogeneous equilibria might be present. Note that in the special case that the kernel is balanced, i.e.  $w_{0}=0$, $\overset{ˆ}{u}=0$ is the only homogeneous equilibrium.

Linearising (1)  +  (2a) around $\overset{ˆ}{u}$ gives

(19) $$\frac{\partial v\left(t,r\right)}{\partial t}=-v\left(t,r\right)+\kappa \int_{\Omega }w\left(r\cdot r^{\prime }\right)v\left(t-\tau \left(r\cdot r^{\prime }\right),r^{\prime }\right)\;dr^{\prime }\text{,}$$

where $\kappa =f^{\prime }\left(\overset{ˆ}{u}\right)$. Solutions of this linear equation are separable and we set $\xi \left(t,r\right)=e^{\mu t}q\left(r\right)$. In this case q(r) satisfies the linear equation Δ(μ)q=0 where

(20) $$\left(\Delta \left(\mu \right)q\right)\left(r\right)≔\left(\mu +1\right)q\left(r\right)-\kappa \int_{\Omega }G\left(r,r^{\prime };\mu \right)q\left(r^{\prime }\right)dr^{\prime }\text{,}$$

is the characteristic function with $G\left(r,r^{\prime };\mu \right)=w\left(r\cdot r^{\prime }\right)\exp\left(-\mu \tau \left(r\cdot r^{\prime }\right)\right)$. Note that the particular structure of G allows application of Theorem 2, which yields coefficients $G_{n}\left(\mu \right)$—see Appendix B. Indeed, Lemma 3, Lemma 4 show that the functions w and τ, [−1,1]→R are Hölder continuous with exponent α, 1/2<α≤1. It is then easily verified that the function [−1,1]→R, $s\to w\left(s\right)e^{-\mu \tau \left(s\right)}$ is also Hölder continuous with exponent α, and the beginning of the proof of Theorem 2 shows that the function Ω×Ω→R, $\left(r,r^{\prime }\right)\to w\left(r\cdot r^{\prime }\right)e^{-\mu \tau \left(r\cdot r^{\prime }\right)}$ is Hölder continuous with exponent α. Now, λ is an eigenvalue of (19) if Δ(λ) has non-trivial solutions. This occurs when

(21) $$E_{n}\left(\lambda \right)≔\lambda +1-\kappa G_{n}\left(\lambda \right)=0\text{,}$$

for some n≥0. In this case, (19) has 2n+1 solutions (i.e. eigenfunctions) of the form $\xi_{m}\left(t,r\right)=e^{\lambda t}Y_{n}^{m}\left(r\right)$, m=−n,…,n. Hence, the algebraic and geometric multiplicity of the eigenvalues are the same.

The spectrum $\sigma ≔\sigma_{p}\cap \sigma_{\mathrm{ess}}$ corresponding to (19) consists of both a point spectrum $\sigma_{p}$ and a (Browder) essential spectrum $\sigma_{\mathrm{ess}}=\left\{-1\right\}$, see also  [29]. The point spectrum consists of all complex numbers which solve (21),

(22) $$\sigma_{p}≔\left\{\lambda \in C|\exists \;n\geq 0:E_{n}\left(\lambda \right)=0\right\}\text{.}$$

We call μ∉σ a *regular value*.

### 3.2 Resolvent

Recall that the point spectrum corresponds to values of λ for which the operator Δ(λ) is not invertible. For all regular values the following theorem holds.

Theorem 3 **Resolvent**

*For given* y∈V *and* μ∉σ*, there exists a unique* q∈V *which solves*

Δ(μ)q=y.

*If both* q *and* y *are expanded in spherical harmonics, see*   Theorem  1*, then coefficients* $q_{n}^{m}$ *are given by*

$$q_{n}^{m}=\frac{y_{n}^{m}}{\mu +1-\kappa G_{n}\left(\mu \right)}\text{.}$$

Proof Existence and uniqueness of the solution are shown in  [29, Prop. 14]. To identify the solution, we start with Δ(μ)q=y, substitute (20) and expand G using Theorem 2

$$\left(\mu +1\right)q\left(r\right)-\overset{\infty }{\underset{n^{\prime }=0}{\sum }}\kappa G_{n^{\prime }}\left(\mu \right)\overset{n^{\prime }}{\underset{m^{\prime }=-n^{\prime }}{\sum }}\int_{\Omega }Y_{n^{\prime }}^{m^{\prime }}\left(r\right)\bar{Y_{n^{\prime }}^{m^{\prime }}\left(r^{\prime }\right)}q\left(r^{\prime }\right)dr^{\prime }=y\left(r\right)\text{,}$$

where integration and summation are interchanged. Next, we multiply both sides by $\bar{Y_{n}^{m}\left(r\right)}$, integrate over the domain w.r.t. r and change the order of integration:

$$\left(\mu +1-\kappa G_{n}\left(\mu \right)\right)\int_{\Omega }q\left(r\right)\bar{Y_{n}^{m}\left(r\right)}dr=\int_{\Omega }y\left(r\right)\bar{Y_{n}^{m}\left(r\right)}dr\text{,}$$

where only one term remains in the summation due to orthonormality of the spherical harmonics. Application of Theorem 1 yields

$$\left(\mu +1-\kappa G_{n}\left(\mu \right)\right)q_{n}^{m}=y_{n}^{m}\text{.}$$

Since μ∉σ, the first factor is non-zero and, hence, we obtain the final identity via division. □

Although we will not use the resolvent in the remainder of this section, it will play a prominent role in the evaluation of the normal form coefficients in Section  5 and Appendix C.

### 3.3 Stability region

The homogeneous steady state $\overset{ˆ}{u}$ is stable if Reλ<0 for all λ∈σ. If real eigenvalues vanish, due to a parameter variation, a fold or transcritical bifurcation will occur. If, instead, eigenvalues have a vanishing real part but a nonzero complex part, then a Hopf bifurcation is expected. In the former case one would expect the formation of time-independent patterns, and in the latter the emergence of travelling or standing waves. Note that in the absence of delays (τ=0), all eigenvalues are real and given explicitly by $\lambda_{n}=-1+\kappa w_{n}$. In this case Hopf bifurcations are not possible. The focus of the remainder of this paper will be on the emergence of spatio-temporal patterns as expected from the general theory of Hopf bifurcations with symmetry  [44], [45].

**Fig. 1:**
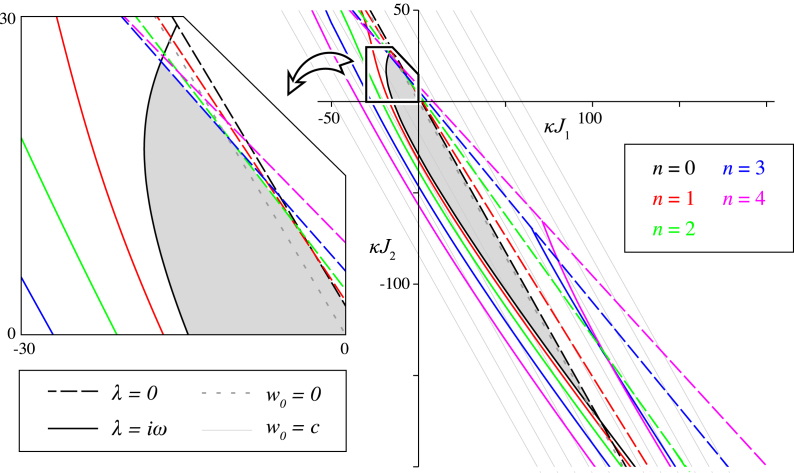
Stability and bifurcations of the homogeneous steady state in the absence of the offset delay $\tau_{0}$. Parametric curves in the $\left(\kappa J_{1},\kappa J_{2}\right)$-plane mark the boundary of the stability region, which is coloured grey. Solid (dashed) coloured lines represent parameters at which the steady state undergoes a Hopf (fold/transcritical) bifurcation with respect to the spherical harmonics of degree n. Grey parallel lines in the background mark lines along which κ is constant. In particular, the line passing through the origin is dashed, which corresponds with a balanced kernel for which explicit calculations can be made. Parameters: $\tau_{0}=0$, c=0.8, $\sigma_{1}=1/3$, $\sigma_{2}=1/4$. (For interpretation of the references to colour in this figure legend, the reader is referred to the web version of this article.)

For the chosen connectivity and delay functions (16), (17) we are able to find explicit expressions for the coefficients $G_{n}\left(\lambda \right)$, of the form

$$G_{n}\left(\lambda \right)=J_{1}h_{n}\left(\lambda ;\sigma_{1}\right)+J_{2}h_{n}\left(\lambda ;\sigma_{2}\right)\text{,}$$

with $h_{n}\left(\lambda ;\sigma \right)$ according to Appendix B. Using (21), one can identify the parameters $\left(J_{1},J_{2}\right)$ at which the homogeneous steady state undergoes a fold or transcritical bifurcation—in this case λ=0. Indeed,

(23) $$1=\kappa J_{1}h_{n}\left(0;\sigma_{1}\right)+\kappa J_{2}h_{n}\left(0;\sigma_{2}\right)\text{,}$$

defines a line in the $\left(\kappa J_{1},\kappa J_{2}\right)$-plane which corresponds to candidate fold/transcritical bifurcations corresponding to the mode number n. In Fig. 1, these lines are plotted with different colours according to their mode number. Similarly, we trace out the candidate Hopf bifurcations by solving (21) for λ=iω,

$$\kappa J_{1}h_{n}\left(i\omega ;\sigma_{1}\right)+\kappa J_{2}h_{n}\left(i\omega ;\sigma_{2}\right)=1+i\omega \text{,}$$

whose solutions are expressed parametrically in terms of ω by equating real and imaginary parts:

(24) [κJ1κJ2](ω)=[Re[hn(iω;σ1)]Re[hn(iω;σ2)]Im[hn(iω;σ1)]Im[hn(iω;σ2)]]−1[1ω].

These parametric curves are plotted for increasing n in Fig. 1 as solid lines.

Furthermore, for small $\kappa J_{1}$ and $\kappa J_{2}$, the linear equation (19) is dominated by the term −v(t,r) such that solutions near the origin of the $\left(\kappa J_{1},\kappa J_{2}\right)$-plane are asymptotically stable. Since instabilities only occur at the curves (23), (24), the stability region can be extended from the origin to the first bifurcation. This region is coloured grey in Fig. 1.

Recall that the fixed points of the system (18) depend on $w_{0}=J_{1}h_{0}\left(0;\sigma_{1}\right)+J_{2}h_{0}\left(0;\sigma_{2}\right)$ and, hence, κ has an implicit dependency on $J_{1}$ and $J_{2}$ as well. As such, the coordinates shown in Fig. 1 are conditional on solutions of the transcendental equation (18). For parameter variations, however, along a line in the set $\left\{J_{1}h_{0}\left(0;\sigma_{1}\right)+J_{2}h_{0}\left(0;\sigma_{2}\right)=C,C\in R\right\}$, the fixed point structure–and therefore κ–remains unaltered. This collection of parallel lines is illustrated in Fig. 1 for various C. One line is of particular importance: the line $w_{0}=0$ corresponds to a balanced kernel, such that $\overset{ˆ}{u}=0$ is the only (homogeneous) fixed point in the system and κ can be determined explicitly.

### 3.4 Remarks

From Fig. 1 we conclude that predominantly spatially homogeneous instabilities are expected to occur, since the stability region is largely bounded by fold/transcritical and Hopf bifurcations corresponding to n=0. Only a small part of the stability region, as shown in the inset, is bounded by curves relating to bifurcations of higher degree in n.

**Fig. 2:**
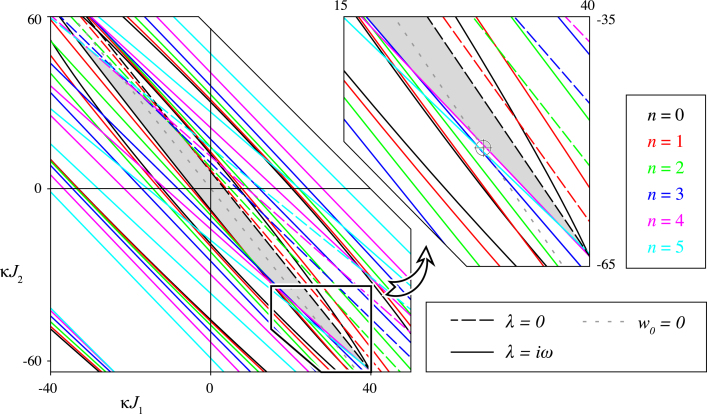
Stability and bifurcations of the homogeneous steady state for non-zero $\tau_{0}$. Similar to Fig. 1, but for different parameters. The parallel lines along which κ is constant are not plotted for clarity. The inset shows a marker at parameter values for which the spectrum is shown in Fig. 3. Parameters: $\tau_{0}=3$, c=0.8, $\sigma_{1}=2/9$, $\sigma_{2}=1/6$. (For interpretation of the references to colour in this figure legend, the reader is referred to the web version of this article.)

Fig. 2 depicts a similar diagram to Fig. 1 for different parameters values. In particular, the offset delay is non-zero. First of all, it is noted that this increment results in a shift of the stability region: the stability region is now positioned more symmetrically around the origin. Furthermore, the Hopf bifurcation curves have a richer structure than in the case where $\tau_{0}=0$, giving rise to many intersections (corresponding to double Hopf bifurcations). As a consequence, the stability region is bounded by curves relating to Hopf bifurcations of spherical harmonics of degrees n≤5. The close-up shows the part of the stability region which is bounded by the Hopf curve for degree n=4.

**Fig. 3:**
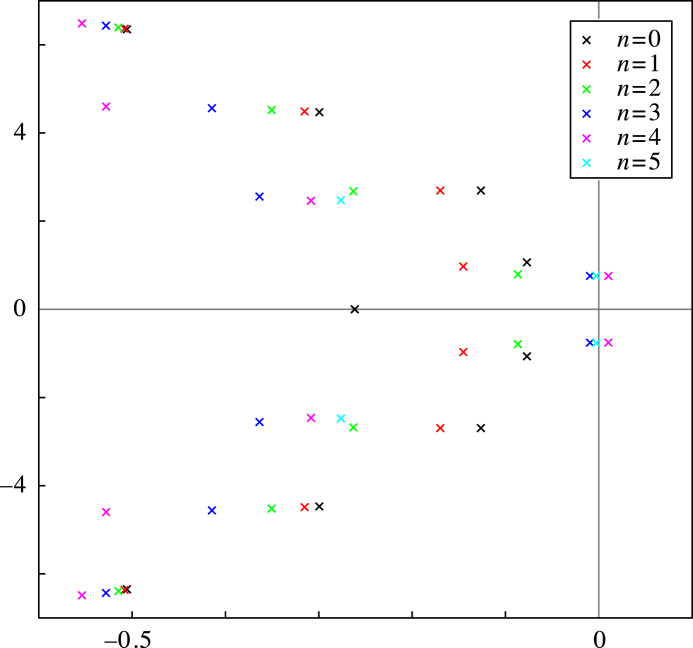
Spectrum of the spatially homogeneous steady state after a Hopf bifurcation. The eigenvalues are determined by solving (20) numerically for n≤5. The pair in the right half-plane corresponds with n=4. Parameters as in Fig. 2 and additionally $\kappa J_{1}=29.50$ and $\kappa J_{2}=-51.38$.

For parameters indicated with the marker in the inset of Fig. 2, we compute the spectrum to verify the foregoing analysis. The result is depicted in Fig. 3 where we show eigenvalues λ=ρ+iω in the (ρ,ω) plane for n≤5, as determined by solving (20) numerically. A pair of complex eigenvalues, corresponding to n=4 can be seen in the positive half-plane, which matches with leaving the stability region by crossing the Hopf curve of the same degree (magenta in Fig. 2).

**Fig. 4:**
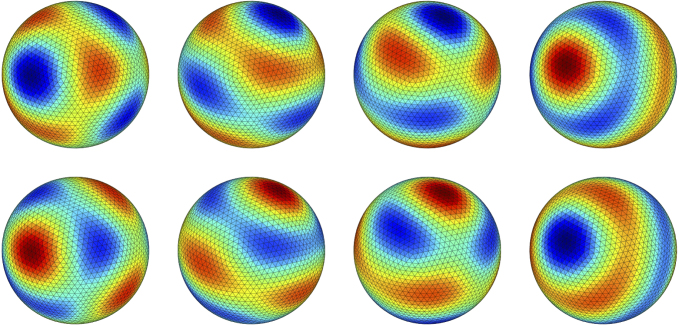
A direct simulation of the spherical Nunez model just beyond the point of an instability with n=4 showing the onset of a standing wave. Left to right, top to bottom shows eight (equally spaced in time) snapshots of the standing wave for one period of oscillation. Warm (cold) colours correspond to high (low) values of u. Parameters as in Fig. 3 and additionally β=8 and δ=0. Simulated with a mesh of 5120 triangles. (For interpretation of the references to colour in this figure legend, the reader is referred to the web version of this article.)

In Fig. 4 we show a direct simulation of an instability to a pattern state with n=4 and ω≠0, highlighting the emergence of a standing wave. Predictions of the bifurcation point as predicted by the linear stability analysis are found to be in excellent agreement with the results from direct numerical simulations in all cases. These were performed using a bespoke numerical scheme, which combines standard techniques for tessellating the sphere with a new approach for solving integro-differential equations with delays on large meshes  [31].

## 4 Intermezzo: planforms

Near a Hopf bifurcation, as identified by the foregoing analysis, the solutions destabilise tangent to the critical eigenspace. Therefore, we expect a dynamical pattern of the form

$$u_{n_{c}}\left(t,r\right)=\overset{n_{c}}{\underset{m=-n_{c}}{\sum }}z_{m}\left(t\right)Y_{n_{c}}^{m}\left(r\right)+\text{cc ,}$$

where cc stands for *complex conjugate*, with $n_{c}$ and $\omega_{c}$ determined from the spectral equation (21) such that $E_{n_{c}}\left(i\omega_{c}\right)=0$ while Reλ<0 for all other λ∈σ. Sufficiently close to the bifurcation point we expect certain classes of solutions to emerge that break the O(3) symmetry of the homogeneous steady state. The tools of equivariant bifurcation theory help to identify solution candidates based on symmetry arguments alone  [44, Chapter XVIII Section 5],  [46]. Typically this is done by developing a system of ordinary differential equations for the evolution of the amplitudes $z=\left(z_{-n_{c}},\ldots ,z_{n_{c}}\right)\in C^{2n_{c}+1}$. If we denote the space spanned by the spherical harmonics of degree n by $V_{n}$ then the action of $O\left(3\right)\times S^{1}$ on $u_{n_{c}}\in V_{n_{c}}⊕V_{n_{c}}$ is determined by its action on z and we will consider $\overset{˙}{z}=f\left(z\right)$, where f is a smooth function that commutes with the action of $O\left(3\right)\times S^{1}$ on $V_{n_{c}}⊕V_{n_{c}}$. By this we mean that f(z) is equivariant with respect to the action of $O\left(3\right)\times S^{1}$ on $V_{n_{c}}⊕V_{n_{c}}$:

(25) $$\gamma \cdot f\left(z\right)=f\left(\gamma \cdot z\right)\;\text{for all }\gamma \in O\left(3\right)\times S^{1}\text{.}$$

In order to determine the structure of the equivariant normal form on $V_{n_{c}}⊕V_{n_{c}}$ to a given order we use the action of elements of $O\left(3\right)\times S^{1}$ on the spherical harmonics of order $n_{c}$ and the raising and lowering operators  [47]. Recall that $O\left(3\right)=SO\left(3\right)⊕Z_{2}^{c}$ and note that the inversion element $-I\in Z_{2}^{c}$ acts on spherical harmonics of degree n as multiplication by ${\left(-1\right)}^{n}$ (for the *natural* action, which is the action which we shall be considering throughout). The group O(3) contains a maximal torus SO(2) corresponding to rotations in the ϕ direction (z-axis) through an arbitrary angle $\bar{\phi }$ which act on $Y_{n}^{m}\left(\theta ,\phi \right)$ as multiplication by $e^{im\bar{\phi }}$. The raising and lowering operators act on the amplitudes $z_{m}$ as  [47]

$$J^{\pm }z_{m}=\sqrt{\left(n_{c}\mp m\right)\left(n_{c}\pm m+1\right)}z_{m\pm 1}\text{.}$$

Symmetry can also be used to determine branches of periodic solutions of $\overset{˙}{z}=f\left(z\right)$. Let, without loss of generality, z(t) be a periodic solution of $\overset{˙}{z}=f\left(z\right)$ with period 2π. Then $\left(\gamma ,\psi \right)\in O\left(3\right)\times S^{1}$ is a spatiotemporal symmetry of z(t) if

(26) (γ,ψ)⋅z(t)≡γ⋅z(t+ψ)=z(t)for all  t.

The set of all spatiotemporal symmetries of a solution z(t) is a subgroup of $O\left(3\right)\times S^{1}$ called the isotropy subgroup of z(t) and denoted $\Sigma_{z\left(t\right)}$. An isotropy subgroup Σ is C-axial if dim Fix(Σ)=2 (i.e. the subspace of $V_{n_{c}}⊕V_{n_{c}}$ which is invariant under the action of Σ is two dimensional). The equivariant Hopf theorem (see  [44]) states that $\overset{˙}{z}=f\left(z\right)$ is guaranteed to have a branch of periodic solutions with symmetry (isotropy) $\Sigma \subset O\left(3\right)\times S^{1}$ bifurcating from the point of dynamic instability if Σ is a C-axial isotropy subgroup of $O\left(3\right)\times S^{1}$ for the action of $O\left(3\right)\times S^{1}$ on $V_{n_{c}}⊕V_{n_{c}}$. This theorem also requires that the eigenvalues cross the imaginary axis with non-zero speed. Thus the symmetries of the branches of periodic solutions bifurcating at a dynamic instability where the modes of degree $n_{c}$ become unstable correspond to the subgroups $\Sigma \subset O\left(3\right)\times S^{1}$ which are C-axial under the action of $O\left(3\right)\times S^{1}$ on $V_{n_{c}}⊕V_{n_{c}}$. Which subgroups are C-axial isotropy subgroups depends on the value of $n_{c}$ and have been determined for all values of $n_{c}$   [44], [45]. The isotropy subgroups of $O\left(3\right)\times S^{1}$ are twisted subgroups

(27) $$H^{\theta }=\left\{\left(h,\theta \left(h\right)\right)\in O\left(3\right)\times S^{1}:h\in H\right\}\text{,}$$

where H is a subgroup of O(3) and $\theta :H\to S^{1}$ is a group homomorphism. Elements of O(3) can be thought of as spatial symmetries whilst elements of $S^{1}$ are temporal symmetries acting on periodic solutions by a phase shift. An element $\left(h,\theta \left(h\right)\right)\in O\left(3\right)\times S^{1}$ is a spatial symmetry if θ(h)=0 and a spatiotemporal symmetry if θ(h)≠0. The spatial symmetries form a normal subgroup K=ker(θ) of H and the quotient group H/K is isomorphic to a closed subgroup of $S^{1}$ (i.e.  1, $Z_{n}\;\left(n\geq 2\right)$ or $S^{1}$). The C-axial isotropy for values of $n_{c}$ between 1 and 6 is given in Table 1 which is reproduced from  [45]. All notations for the subgroups of O(3) here and throughout are consistent with that in  [44], [45].

**Fig. 5:**
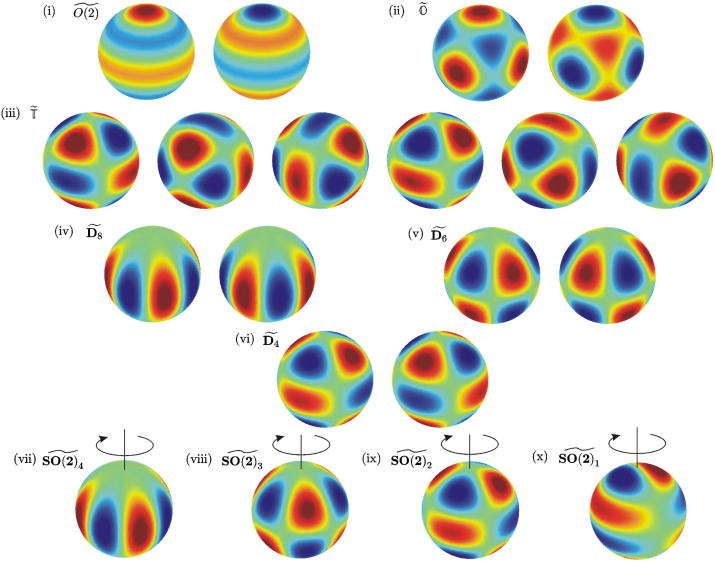
The ten periodic solutions guaranteed to exist at a dynamic instability with $n_{c}=4$ corresponding to axial isotropy subgroups as listed in Table 2. (i)–(vi) illustrate the evolution of the six standing wave solutions over one period and (vii)–(x) illustrate the travelling wave solutions indicating the apparent axis and direction of rotation.

Observe for example that if the spherical harmonics of degree $n_{c}=4$ become unstable at the dynamic instability, then branches of periodic solutions with at least ten distinct symmetry types bifurcate. These solutions are listed in Table 2 and illustrated in Fig. 5. There are six standing wave solutions (where the image of subgroup H⊂O(3) under the homomorphism θ is 1 or $Z_{n}\;\left(n\geq 2\right)$) and four travelling wave solutions (which have $\theta \left(H\right)=S^{1}$ since the spatial symmetry of a travelling wave does not change over time). Note from Table 2 that the travelling wave solutions consist of a single spherical harmonic rotating in one direction whereas standing wave solutions result from the sum of spherical harmonics $Y_{n_{c}}^{m}$ and $Y_{n_{c}}^{-m}$. The resulting standing wave can be thought of as arising due to interference between the waves travelling in opposite directions around the sphere.

## 5 Intermezzo: centre manifold reduction

In order to determine which patterns are stable near the bifurcation, we apply the method developed in  [29], which provides a generic method for normal form computation in delayed neural fields. The functional analytic setting of this work is based on sun–star calculus, which is described in  [48] for traditional delay differential equations. Since the forementioned works are particularly technical, we aim to offer the reader a *rudimentary overview* of the sun–star framework leaving out many details; these can be found in the original works. In particular, we will introduce and discuss all components of Eqs. (35a)  +  (35b), which are required to compute the critical normal form coefficients of bifurcations.

### 5.1 Sun-star calculus

The space X is the state space of the delayed equation (1) and elements $x_{t}\in X$ relate to the system’s history via $x_{t}\left(\theta \right)≔x\left(t+\theta \right)\;\forall t\geq 0,\theta \in \left[-h,0\right]$. Next, consider the linear system

(28) $$\left\{ \begin{array}{cc} \overset{˙}{x}\left(t\right)=Lx_{t} & t\geq 0\text{,}\\ x\left(t\right)=\eta \left(t\right) & t\in \left[-h,0\right]\text{,} \end{array}\right.$$

with initial condition η∈X and linear operator L:X↦V. For our particular system, L is given by^1^

(29) $$\left(L\eta \right)\left(r\right)=-\eta \left(0,r\right)+\kappa \int_{\Omega }w\left(r\cdot r^{\prime }\right)\eta \left(-\tau \left(r\cdot r^{\prime }\right),r^{\prime }\right)dr^{\prime }\;\forall \eta \in X,\forall r\in \Omega \text{.}$$

Note that, in general, the evolution of the state $x_{t}\in X$ of a time-delayed system involves two actions. The first equation of (28) is a rule for the *extension* to the future. Secondly, the present state *shifts* through the history as time progresses:

$$x_{t+\Delta t}\left(\theta \right)=x_{t}\left(\theta +\Delta t\right)\;\theta \in \left[-h,0\right],\;\Delta t\geq 0:-h\leq \theta +\Delta t\leq 0\text{.}$$

Formally, consider the strongly continuous semigroup T(⋅), which solves (28), that is $x_{t}=T\left(t\right)\eta ,\forall t\geq 0$. Associated with T is the abstract differential equation

$$\frac{d}{dt}\left(T\left(t\right)\eta \right)=A\left(T\left(t\right)\eta \right)\text{,}$$

where A:D(A)⊂X↦X is the generator defined as

$$A\eta =\underset{t↓0}{\lim}\frac{1}{t}\left(T\left(t\right)\eta -\eta \right)\text{,}$$

and corresponding domain D(A) such that this limit exists. Hence,

(30) $$A\eta =\overset{˙}{\eta }\;\text{and}\;D\left(A\right)=\left\{\eta \in X:\overset{˙}{\eta }\in X\text{ and }\overset{˙}{\eta }\left(0\right)=L\eta \right\}\text{.}$$

With the generator in this form, we can indeed see that the solution is generated by shifting and extending. Namely, the action of A is differentiation, which is the generator of the shift semigroup $T_{0}$. The extension component, on the other hand, is incorporated in the domain of A, suggesting that the solution of (28) is generated by shifting only those functions that satisfy the differential equation. Here, we stress the fact that the appearance of the differential equation as a condition on the domain is cumbersome. If we, at this point, were to proceed with a linear parameter-dependent perturbation of the differential equation, the domains of definition of the solution spaces would change, obstructing standard bifurcation analysis.

This particular problem is overcome if we study the problem in a new and ‘larger’ space $X^{⊙*}$. The space $X^{⊙*}$ (pronounced: sun–star) is best thought of as the double dual space of X with additional canonical restrictions geared towards maintaining strong continuity of the semigroup. In our case $X^{⊙*}=V^{**}\times {\left[L^{1}\left(\left[0,h\right];V^{*}\right)\right]}^{*}$, which cannot be represented in terms of known functions or measures. Yet, X is canonically embedded in $X^{⊙*}$ via $j:X\mapsto X^{⊙*}$ given by jη=(η(0),η)—here we exploited the fact that $V\times L^{\infty }\left(\left[-h,0\right];V\right)\subset X^{⊙*}$, which suffices for our purpose.

The flow on $X^{⊙*}$ is generated by $A^{⊙*}$. If $\eta \in C^{1}\left(\left[-h,0\right];V\right)\subset X$, then $j\eta \in D\left(A^{⊙*}\right)$ and $A^{⊙*}j\eta =\left(L\eta ,\overset{˙}{\eta }\right)$. Comparing $A^{⊙*}$ with A as in (30), we see that the condition on the domain, which deals with the right hand side of the differential equation, is transformed into an action of the operator (i.e. the first component of $A^{⊙*}j\eta$). In this setting, we can readily perturb the linear system (28) without altering the space $X^{⊙*}$ or the domain of $A^{⊙*}$.

### 5.2 Centre manifold and homological equation

We proceed to the non-linear model which generalises the neural field (1)  +  (2a):

(31) $$\left\{ \begin{array}{cc} \overset{˙}{x}\left(t\right)=F\left(x_{t}\right) & t\geq 0\text{,}\\ x\left(t\right)=\eta \left(t\right) & t\in \left[-h,0\right]\text{,} \end{array}\right.$$

for some Lipschitz continuous F:X↦V. Note that the differential equation is an equation in $V\subset V^{**}$ and, upon assuming $x_{t}\in C^{1}\left(\left[-h,0\right];V\right),\forall t\geq 0$, we can extend (31) to an equation in $X^{⊙*}$

$$\underset{︸}{\left[\begin{array}{c} {\overset{˙}{x}}_{t}\left(0\right)\\ {\overset{˙}{x}}_{t} \end{array}\right]}=\underset{︸}{\left[\begin{array}{c} Lx_{t}\\ {\overset{˙}{x}}_{t} \end{array}\right]}+\underset{︸}{\left[\begin{array}{c} F\left(x_{t}\right)-Lx_{t}\\ 0 \end{array}\right]}$$

(32) $$j{\overset{˙}{x}}_{t}=A^{⊙*}jx_{t}+\;R\left(x_{t}\right)\text{.}$$

The last equation defines an abstract differential equation on $X^{⊙*}$, where $R:X\mapsto X^{⊙*}$ is a true non-linearity in its first component.

Whenever the system (31) is at a critical point, i.e.  L has N eigenvalues with vanishing real part, then there exists a locally invariant centre manifold $W^{c}$, which is tangent to the critical eigenspace at the origin. Moreover, if there are also N critical eigenfunctions $\xi_{i}$, then there exists a smooth $H:W\subset R^{N}\mapsto X$ such that $H\left(W\right)=W^{c}$. We expand H

$$H\left(z\right)=\overset{N}{\underset{i=1}{\sum }}z_{i}\xi_{i}+\underset{2\leq \left|\nu \right|\leq 3}{\sum }\frac{1}{\nu !}h_{\nu }z^{\nu }+O\left({\left|z\right|}^{4}\right)\text{,}$$

where ν is a multi-index of length N and $h_{\nu }\in X$. (Note that, in the case of complex eigenvalues, the expansion is usually chosen differently; see Appendix C.) On the centre manifold, the system satisfies some ODE in $R^{N}$ that is equivalent to the normal form

(33) $$\overset{˙}{z}\left(t\right)=\underset{1\leq \left|\nu \right|\leq 3}{\sum }g_{\nu }z^{\nu }+O\left({\left|z\left(t\right)\right|}^{4}\right)\text{,}$$

with unknown critical normal form coefficients $g_{\nu }\in R^{N}$. Due to invariance of the centre manifold, we can restrict the dynamics to the centre manifold, i.e.  $x_{t}=H\left(z\left(t\right)\right)$, and substitute (32) to obtain the homological equation

(34) $$jDH\left(z\right)\overset{˙}{z}=A^{⊙*}jH\left(z\right)+R\left(H\left(z\right)\right)\text{.}$$

Next, R admits the expansion

$$R\left(\eta \right)=\frac{1}{2!}B\left(\eta ,\eta \right)+\frac{1}{3!}C\left(\eta ,\eta ,\eta \right)+O\left({\left\|\eta \right\|}^{4}\right)\text{,}$$

where B and C are the bi- and tri-linear operators corresponding respectively to the second and third derivatives of F, cf. Appendix C.1. Note that, since R is zero in its second component, also B and C will be zero in their second components.

Finally, (34) allows us to find expressions for the critical normal coefficients. Equating coefficients of powers of z on both sides, one recursively obtains expressions for $h_{\nu }$ and $g_{\nu }$. In particular, one encounters linear systems of two forms which can be solved explicitly:

• For μ a regular value (i.e.  μ∉σ(A)), η∈X and v∈V
  (35a) $$\left(\mu -A^{⊙*}\right)j\eta =\left(v,0\right)⟹\eta \left(t\right)=e^{\mu t}\Delta^{-1}\left(\mu \right)v$$
  with $\Delta^{-1}\left(\mu \right)$ the resolvent as in Theorem 3.
• For λ an eigenvalue of L (i.e.  λ∈σ(A)), with corresponding eigenfunction $\xi \left(t,r\right)=e^{\lambda t}q\left(r\right)\in X$, v∈V and ℓ∈C, Fredholm solvability yields a condition for ℓ
  (35b) $$\left(\lambda -A^{⊙*}\right)j\eta =\left(v,0\right)+\ell j\xi ⟹\ell q=-\frac{1}{2\pi i}\oint_{\partial C_{\lambda }}\Delta^{-1}\left(\mu \right)v\;d\mu \text{,}$$
  where $C_{\lambda }\subset C$ is a punctured disk around λ, with sufficiently small radius such that $C_{\lambda }\cap \sigma \left(A\right)=\emptyset$.

We note that the two foregoing statements are not explicitly stated in the original works  [29], [30], but are discussed and implemented in §4.4 and §4.3 respectively.

## 6 Bifurcations and normal form computation

In this section we combine the various results from the foregoing sections to identify and classify several bifurcations in the model (1)  +  (2a). Using the stability results from Section  3, we identify the precise parameter values of the bifurcation points. Since we know the mode number(s) involved in the bifurcation, the planforms in Section  4 inform us about the relevant symmetries and the corresponding normal form equation. This normal form is, in turn, at the heart of the centre manifold reduction reviewed in Section  5, which enables us to computate the relevant coefficients in the normal form. All that remains at this point, is to analyse the behaviour of the normal form’s low-dimensional dynamics. While these have been studied and documented in great detail for bifurcations of codimension 1 and 2 in systems without symmetry, e.g.  [49], the normal forms of symmetric bifurcations are numerous and, therefore, lacking a clear overview. As a consequence, we will devote part of this section to the analysis of the low-dimensional system that arises from a double Hopf bifurcation with a special symmetry.

More concretely, we study the following two bifurcations:

• Hopf bifurcation where $E_{0}\left(i\omega_{0}\right)=0$, and
• double Hopf bifurcation for mixed interactions, where $E_{0}\left(i\omega_{0}\right)=0$ and $E_{1}\left(i\omega_{1}\right)=0$.

A third case, the Hopf bifurcation due to $E_{1}$ only, is treated in Appendix C.3.

### 6.1 Hopf bifurcation, $E_{0}$

As a starting point we will analyse the simplest Hopf bifurcation in the system, namely the one without symmetry. The reasons for this are twofold: Firstly, Fig. 1 reveals that for $\tau_{0}=0$ the stability region is largely bounded by the Hopf bifurcation corresponding to n=0. Secondly, the centre manifold is more accessible because we can apply the results for the generic Hopf bifurcation given in  [29]. We are, however, in a better position than the authors of the original work: since our model allows its eigenfunctions to be expressed analytically, we are able to find a closed expression of the first Lyapunov coefficient $l_{1}$.

If for $\omega_{0}>0$, $i\omega_{0}$ is a simple root of $E_{0}$ and $E_{n}\left(i\omega \right)\neq 0$ for all other n and ω≥0, then the system undergoes a Hopf bifurcation w.r.t. the mode n=0. In this case, the eigenvalue $\lambda =i\omega_{0}$ has both algebraic and geometric multiplicity equal to 1, and its eigenfunction is $\xi \left(t,r\right)=e^{i\omega_{0}t}Y_{0}^{0}\left(r\right)=e^{i\omega_{0}t}/\sqrt{4\pi }$. Since the eigenfunction is constant across space, the oscillations originating from this bifurcation will also be homogeneous across space. Therefore, we denote this pattern as a *bulk oscillation*.

Because the eigenvalue has multiplicity one, the normal form for this Hopf bifurcation is given by:

(36) $$\overset{˙}{z}=\left(\rho_{0}+i\omega_{0}\right)z+g_{21}z^{2}\bar{z}\text{,}$$

with $g_{21}$ the coefficient determining the criticality. Here $\rho_{0}$ denotes the real part of the critical eigenvalue in the neighbourhood (in parameter space) of the bifurcation; clearly $\rho_{0}=0$ at the bifurcation. Moreover, the *first Lyapunov coefficient* is defined as l1≔1ω0Reg21. Using the techniques described in Section  5, we are able to find an explicit expression for this coefficient (cf. Appendix C.2):

(37) $$g_{21}=\frac{1+i\omega_{0}}{8\pi \kappa \left(1-\kappa G_{0}^{\prime }\left(i\omega \right)\right)}\left(f^{\prime \prime \prime }\left(\overset{ˆ}{u}\right)+f^{\prime \prime }{\left(\overset{ˆ}{u}\right)}^{2}\left[Q_{0}\left(2i\omega_{0}\right)+2Q_{0}\left(0\right)\right]\right)\text{,}$$

where

(38) $$Q_{n}\left(\mu \right)≔\frac{G_{n}\left(\mu \right)}{\mu +1-\kappa G_{n}\left(\mu \right)}\text{.}$$

Now, a negative (positive) sign of the first Lyapunov coefficient corresponds with a supercritical (subcritical) bifurcation.

**Fig. 6:**
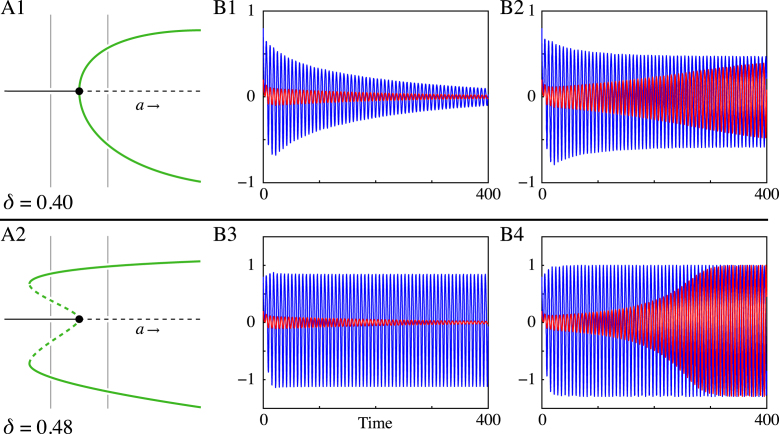
Generalised Hopf bifurcation occurs for δ≈0.4172 and α=1. (A1) shows that for δ=0.40 the Hopf bifurcation is supercritical. Time series for two different initial conditions (red and blue) reveal one stable focus for α=0.98 (B1) while for α=1.02 a stable limit cycle is observed (B2). For δ=0.48, the criticality of the Hopf bifurcation has changed, resulting in a multistable regime (A2). Indeed, in (B3) simulations show that for α=0.98 there exist two stable solutions: a stable focus (red) and a stable limit cycle (blue). For α=1.02, the stable focus has destabilised and only the stable limit cycle remains. Parameter values: β=4, $\tau_{0}=3$, c=1, $\sigma_{1}=1$ and $\sigma_{2}=1/2$, $\kappa J_{1}\approx 1.565$, $\kappa J_{2}\approx -4.075$ and $\omega_{0}\approx 0.950$. (For interpretation of the references to colour in this figure legend, the reader is referred to the web version of this article.)

Using the parametric expression for the Hopf bifurcations in parameter space, as formulated in Section  3, and the choice of f as in (15), we are able to determine the first Lyapunov coefficient along these curves. In particular, we identify parameters for which the first Lyapunov coefficient vanishes, corresponding to a *generalised Hopf bifurcation*. Although we do not compute the second Lyapunov coefficient, we investigate this bifurcation numerically; see Fig. 6. The bistability, as observed in Fig. 6 (B3), between a focus (red) and a limit cycle (blue) suggests that the second Lyapunov coefficient is negative. Note that, since the pattern is homogeneous across space, it suffices to plot time series at only one point on the sphere.

### 6.2 Double Hopf bifurcation, $E_{0}$ and $E_{1}$

If we, starting at the supercritical Hopf bifurcation for n=0, vary a different parameter, we will not observe a change of sign of the first Lyapunov exponent. Instead, we find that another pair of eigenvalues, corresponding to n=1, passes through the imaginary axis. We analyse this double Hopf bifurcation using symmetry techniques.

For n=0,1, let $\omega_{n}>0$ be such that $i\omega_{n}$ is a simple root of $E_{n}$ and, moreover, $E_{n}\left(i\omega \right)$ has no other roots for ω≥0 and n≥0. There is one eigenfunction $\psi \left(t,r\right)=e^{i\omega_{0}t}Y_{0}^{0}\left(r\right)$ related to $i\omega_{0}$, while there are three corresponding to $i\omega_{1}$, namely $\xi_{m}\left(t,r\right)=e^{i\omega_{1}t}Y_{1}^{m}\left(r\right)$ for m=−1,0,1.

In the non-resonant case, i.e.  $k\omega_{0}\neq l\omega_{1}$ for all k,l∈N with k+l≤5, symmetry arguments yield the truncated normal form up to cubic order:

(39a) $$\overset{˙}{w}=\left(\rho_{0}+i\omega_{0}\right)w+a_{1}w{\left|w\right|}^{2}+a_{2}w{\left|z\right|}^{2}\text{,}$$

(39b) $${\overset{˙}{z}}_{m}=\left(\rho_{1}+i\omega_{1}\right)z_{m}+b_{1}z_{m}{\left|z\right|}^{2}+b_{2}{\overset{ˆ}{z}}_{m}\left(z_{0}^{2}-2z_{-1}z_{1}\right)+b_{3}z_{m}{\left|w\right|}^{2}\text{,}\;m=0,\pm 1\text{,}$$

where

(40) $$z≔\left(z_{-1},z_{0},z_{1}\right)\text{,}\;\overset{ˆ}{z}≔\left(-\bar{z_{1}},\bar{z_{0}},-\bar{z_{-1}}\right)\text{,}\;{\left|z\right|}^{2}≔\overset{1}{\underset{m=-1}{\sum }}{\left|z_{m}\right|}^{2}\text{.}$$

Expressions for the critical coefficients, $a_{1}$, $a_{2}$, $b_{1}$, $b_{2}$, $b_{3}$ of the model (2b) are given in Appendix C.4. Moreover, setting $z=\left(w,z_{-1},z_{0},z_{1}\right)$ gives the centre manifold:

(41) $$H\left(z,\bar{z}\right)=w\psi +\bar{w\psi }+\overset{1}{\underset{m=-1}{\sum }}z_{m}\xi_{m}+\bar{z_{m}\xi_{m}}+\underset{\left|\nu \right|+\left|\zeta \right|\geq 2}{\sum }\frac{1}{\nu !\zeta !}h_{\nu \;\zeta }z^{\nu }{\bar{z}}^{\zeta }\text{.}$$

It is important to stress the fact that, since we are neglecting terms of order 4 and higher, we can only study the ‘simple’ case  [49, Section 8.6.2]. Therefore, our analysis is only valid whenever Rea1Reb1>0.

We continue to identify possible solutions of (39). By computing all isotropy subgroups of $O\left(3\right)\times S^{1}$ for the representation on $\left(V_{0}⊕V_{0}\right)⊕\left(V_{1}⊕V_{1}\right)$ we find that there are three C-axial subgroups which are numbered 1–3 in Table 3. This table gives all isotropy subgroups, Σ, for this representation, along with a representative fixed point subspace, Fix(Σ), for one particular choice of set of generators of the subgroup. Branches of solutions with these symmetry types are guaranteed to exist by the equivariant Hopf theorem. The patterns corresponding to solution types 1–3 are respectively bulk oscillations, travelling waves and standing waves.

Other solutions to (39) may exist depending on the values of parameters. These correspond to isotropy types which have fixed point subspaces of dimension greater than 2 and are types 4–8 in Table 3. We make the following observations about the solutions with such symmetries. We have two types of solutions with only n=1 modes (using the numbering in Table 3):

4. This solution has contributions from two different n=1 modes. If a solution with this symmetry were to exist generically (i.e. when Reb2≠0) it would have $z_{-1}=R\left(t\right)e^{i\phi \left(t\right)}$ and $z_{1}=R\left(t\right)e^{i\left(\phi \left(t\right)+\psi \right)}$ for some fixed phase shift ψ.
5. This solution has contributions from two different n=1 modes. Thus $w=0,z=\left(R_{-1}\left(t\right)e^{i\phi_{-1}\left(t\right)},R_{0}\left(t\right)e^{i\phi_{0}\left(t\right)},R_{1}\left(t\right)e^{i\phi_{1}\left(t\right)}\right)$. In this case the equations for the amplitudes $R_{m}\left(t\right)$ and phases $\phi_{m}\left(t\right)$, m=−1,0,1 do not decouple. However the equations have effective dimension 4 since they only depend on the combination of phases $2\phi_{0}-\phi_{1}-\phi_{-1}$.

There could also exist three types of mixed mode solutions. These can only have spatial symmetries since we assume no resonance between the n=0 and n=1 modes.

6. Removing the time shift symmetries of the standing wave solution with $\overset{˜}{O\left(2\right)}$ symmetry, we are left only with the $O{\left(2\right)}^{-}$ symmetry which allows for a nonzero amplitude of the n=0 mode.
7. Solutions with just a reflection symmetry include (for the case of reflection in the xy plane) $w=r\left(t\right)e^{i\theta \left(t\right)},z=\left(R_{-1}\left(t\right)e^{i\phi_{-1}\left(t\right)},0,R_{1}\left(t\right)e^{i\phi_{1}\left(t\right)}\right)$. In this case the equations for the amplitudes again decouple but a solution with $R_{-1}\neq 0,R_{1}\neq 0$ and r≠0 only exists generically in the case that $R_{-1}=R_{1}$ and $\phi_{-1}=\phi_{1}+\psi$ for a constant phase shift ψ.
8. For solutions with no symmetry we have
  $$w=r\left(t\right)e^{i\theta \left(t\right)}\text{,}\;z=\left(R_{-1}\left(t\right)e^{i\phi_{-1}\left(t\right)},R_{0}\left(t\right)e^{i\phi_{0}\left(t\right)},R_{1}\left(t\right)e^{i\phi_{1}\left(t\right)}\right)\text{.}$$
  Here the amplitude and phase equations are coupled but as for solution type 5 they depend only on θ and $2\phi_{0}-\phi_{1}-\phi_{-1}$ so the effective dimension is 6 rather than 8.

Although it is not possible to decouple the system in its original form, a different set of coordinates does. Indeed, the transformation $\left(x_{1},x_{2},x_{3}\right)=\left({\left|w\right|}^{2},\left|z_{0}^{2}-2z_{-1}z_{1}\right|,{\left|z\right|}^{2}\right)\in R^{3}$ yields a decoupled system of ODEs:

(42a) $${\overset{˙}{x}}_{1}=2x_{1}\left(\rho_{0}+{\overset{˜}{a}}_{1}x_{1}+{\overset{˜}{a}}_{2}x_{3}\right)$$

(42b) $${\overset{˙}{x}}_{2}=2x_{2}\left(\rho_{1}+\left({\overset{˜}{b}}_{1}+{\overset{˜}{b}}_{2}\right)x_{3}+{\overset{˜}{b}}_{3}x_{1}\right)$$

(42c) $${\overset{˙}{x}}_{3}=2x_{3}\left(\rho_{1}+{\overset{˜}{b}}_{1}x_{3}+{\overset{˜}{b}}_{3}x_{1}\right)+2{\overset{˜}{b}}_{2}x_{2}^{2}\text{,}$$

where ${\overset{˜}{a}}_{i}$ and ${\overset{˜}{b}}_{i}$ denote the real parts of $a_{i}$ and $b_{i}$ respectively. Fixed points of the system x can be related to all of the eight cases described above; Table 3 shows this relation.

**Fig. 7:**
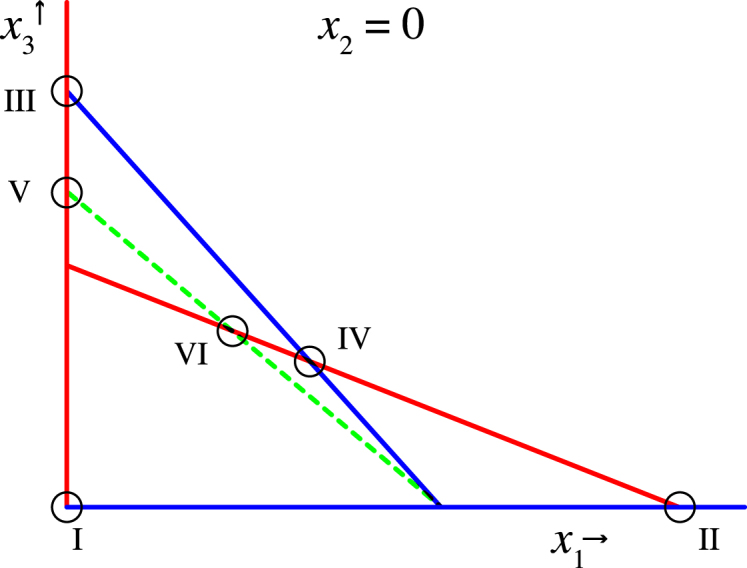
Nullplanes of (42) in the $x_{2}=0$ plane, depicting $x_{1}$, $x_{2}$ and $x_{3}$ in red, green and blue respectively. Note that the plane $x_{2}=0$ is a $x_{2}$-nullplane, such that shown intersections of $x_{1}$- and $x_{3}$-nullplanes correspond with steady states; i.e. points i–iv. For $x_{2}>0$, the $x_{2}$-nullplane is given by the dashed green line, yielding two more equilibria in the unseen dimension: v and vi, for which $x_{2}=x_{3}$. Parameters as follows: $\mu_{1},\mu_{2}>0$ and ${\overset{˜}{a}}_{i},{\overset{˜}{b}}_{j}<0$ for all i,j. (For interpretation of the references to colour in this figure legend, the reader is referred to the web version of this article.)

It is straightforward to show that, generically, the system (42) has at most 6 equilibria in the first octant $x_{1},x_{2},x_{3}\geq 0$. (Note that solutions outside the first octant are irrelevant, for $x_{i}$ are non-negative amplitudes.) For parameters corresponding with the double Hopf bifurcation in the full non-linear model (2a), we find that the real parts of all normal form coefficients in (39) are negative. The phase plane of the corresponding reduced system (42) is shown in Fig. 7, which reveals the occurrence of all 6 steady states. Continuing with a linear stability analysis of these six equilibria, we find that only points ii and iii are asymptotically stable (i.e.  $x=\left(-\mu_{1}/a_{1},0,0\right)$ and $x=\left(0,0,-\mu_{2}/b_{1}\right)$ respectively). Therefore, we conclude from Table 3 the existence of a multistable regime in which we observe either bulk oscillations, travelling waves, or a mixture of different 1-modes (cases 1, 2 and 5 respectively).

**Fig. 8:**
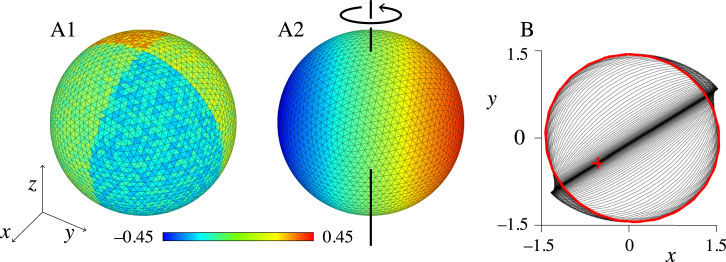
Emergence of a rotating wave in the presence of multistability. Direct simulation of the initial condition A1 for t≤0 evolves into the rotating wave A2. The time-course of the sphere’s centre of mass is shown in B, projected in the x,y-plane. The initial condition, marked with the red cross, is near the (asymptotically) unstable standing wave pattern, causing the system to remain close to this pattern—as is shown by the centre of mass moving oscillating in an almost straight line. In the course of the simulation, the centre of mass approaches its limit cycle (red circle), which corresponds to the rotating wave solution. This stable solution co-exists with stable bulk oscillations (not shown). Parameter values: α=1.08, β=4, δ=0.1, $\tau_{0}\approx 3.483$, c=1, $\sigma_{1}=1$ and $\sigma_{2}=1/2$, $J_{1}\approx 1.678$, $J_{2}\approx -4.367$, $\omega_{0}\approx 0.861$ and $\omega_{1}\approx 0.609$. Simulated with a mesh of 5120 triangles. (For interpretation of the references to colour in this figure legend, the reader is referred to the web version of this article.)

This result is confirmed using direct simulations of the model near the bifurcation. We find two stable solutions, namely bulk oscillations (case 1) and rotating waves (case 2); the latter is depicted in Fig. 8. We have not identified a stable solution consisting of mixed 1-modes (case 5).

## 7 Discussion

In this paper we have analysed pattern formation in a spherical model of brain activity and shown how a rich repertoire of spatio-temporal states can be supported in neural models of Nunez type that contain only simple representations for anatomical connectivity, axonal delays and population firing rates. Even though these models are naturally formulated as nonlinear evolution equations of integro-differential form with delays, which have been little studied, they are amenable to similar analyses used for studying pattern-formation in PDEs. Perhaps surprisingly this is the first time that a combination of linear stability analysis, symmetric bifurcation theory, centre manifold reduction and direct numerical simulations have been combined to explore such a popular model for EEG generation. Most certainly this is because it has proved easier to study versions of the model on the line  [50] or the plane  [51], despite the obvious motivation to study the model on more brain like topologies.

Importantly, our study is the first one to carry out a detailed centre manifold reduction on the full integral formulation of the model, including transmission delays, in a two-dimensional setting—the one-dimensional case is discussed in  [28], [29], [30]. As a result, this new work has shed light on the importance of delays in generating patterns with a high degree of spatial structure, as well as developed the bifurcation theory that can be used to ascertain the emergence of a given symmetric structure via the destabilisation of a homogeneous steady state. Our approach has also allowed us to explicitly pin-point the conditions for codimension-2 bifurcations, where two or more distinct patterns can be excited and then subsequently interact.

Secondary bifurcations, especially those related to multi-stability, have received considerable attention in the modelling of EEG; especially in the context of epileptic seizures. The generalised Hopf bifurcation, with bistability between rest and oscillation, has a pivotal position in explaining ictal transitions in models of cortical columns  [52], [53]. Since these models have been studied primarily in the absence of a spatial component, it has remained unclear what the impact of both spatial structure and transmission delays would be on oscillations and synchrony in the model. In this article we have shown that the generalised Hopf bifurcation can still occur in the extended setting, enabling the model to switch between rest and a fully synchronous periodic solution. The double Hopf bifurcation, which yields a type of multistability where multiple stable periodic solutions can exist, was studied in a neural field context as early as 1980  [54]. Therein, the authors emphasised that the appearance of quasi-periodic behaviour, which occurs in a special case of the bifurcation as a result of mixing between two stable oscillations, could explain the transition between the tonic and clonic seizure states. Being largely theoretical, their work does not provide a methodology for computing or classifying these transitions. Although we have been able to identify these quasi-periodic oscillations in our work (points IV and VI in Fig. 7), we have been unable to find parameters for which they would be stable—it remains an open question whether such parameters exist for our model. Other work on secondary bifurcations in one-dimensional neural field models can be found in  [55].

Of course, the Nunez model is also able to generate a whole host of more exotic behaviour in regimes where our analytical approaches have less sway. For example, Fig. 8 shows the emergence of a large scale rotating wave which is reminiscent of those reported in EEG studies of schizophrenic patients  [56]. In these instances the development of our bespoke numerical scheme pays further dividends since it is not restricted to perfectly spherical models. The scheme is sufficiently general to handle real folded cortical structures with more detailed white matter fibre tractography data, of the type that is increasingly available in public repositories such as the Human Connectome Project  [57]. Indeed one natural extension of the work presented in this paper is the development of a primarily computational model of brain activity that can incorporate more biological detail, such as folded cortical hemispheres  [58], heterogeneous connection topologies  [59] and a distribution of axonal speeds  [60]. Such a model is relevant to interpreting modern whole brain neuroimaging signals, and its exploration would set the scene for formulating the relevant mathematical questions about how best to understand the behaviour of a complex brain model. For example, it would be interesting to explore how wave dispersion and interaction on a folded cortex affects the frequency of emergent rhythms and the dependence of these on underlying activity that is primarily either in the form of travelling waves or standing waves. Even before developing such a programme the model explored here has other solutions that are of interest to the neuroscience community in the form of localised states (such as spots), often invoked in the context of working memory and easily established for a steep sigmoidal firing rate and Mexican-hat connectivity, see for example  [61]. Once again progress in this arena might take as a starting point ideas recently developed for the study of spots for PDEs on a sphere  [62]. These and other topics, including the response of the model to forcing, will be reported upon elsewhere.

## Figures and Tables

**Table 1:** The C-axial subgroups of $O\left(3\right)\times S^{1}$ for the natural representations on $V_{n_{c}}⊕V_{n_{c}}$ for $1\leq n_{c}\leq 6$. Here $H=J\times Z_{2}^{c}$.

$n_{c}$	J	K	θ(H)	Number of branches given by equivariant Hopf theorem
1	O(2)	$O{\left(2\right)}^{-}$	$Z_{2}$	2
	SO(2)	$Z_{2n}^{-}$	$S^{1}\;\left[n=1\right]$	
2	O(2)	$O\left(2\right)\times Z_{2}^{c}$	1	5
	SO(2)	$Z_{n}\times Z_{2}^{c}$	$S^{1}\;\left[n=1,2\right]$	
	T	$D_{2}\times Z_{2}^{c}$	$Z_{3}$	
	$D_{4}$	$D_{2}\times Z_{2}^{c}$	$Z_{2}$	
3	O(2)	$O{\left(2\right)}^{-}$	$Z_{2}$	6
	SO(2)	$Z_{2n}^{-}$	$S^{1}\;\left[1\leq n\leq 3\right]$	
	O	$O^{-}$	$Z_{2}$	
	$D_{6}$	$D_{6}^{d}$	$Z_{2}$	
4	O(2)	$O\left(2\right)\times Z_{2}^{c}$	1	10
	SO(2)	$Z_{n}\times Z_{2}^{c}$	$S^{1}\;\left[1\leq n\leq 4\right]$	
	O	$O\times Z_{2}^{c}$	1	
	T	$D_{2}\times Z_{2}^{c}$	$Z_{3}$	
	$D_{8}$	$D_{4}\times Z_{2}^{c}$	$Z_{2}$	
	$D_{6}$	$D_{3}\times Z_{2}^{c}$	$Z_{2}$	
	$D_{4}$	$D_{2}\times Z_{2}^{c}$	$Z_{2}$	
5	O(2)	$O{\left(2\right)}^{-}$	$Z_{2}$	11
	SO(2)	$Z_{2n}^{-}$	$S^{1}\;\left[1\leq n\leq 5\right]$	
	T	$D_{2}$	$Z_{6}$	
	$D_{10}$	$D_{10}^{d}$	$Z_{2}$	
	$D_{8}$	$D_{8}^{d}$	$Z_{2}$	
	$D_{6}$	$D_{6}^{d}$	$Z_{2}$	
	$D_{4}$	$D_{4}^{d}$	$Z_{2}$	
6	O(2)	$O\left(2\right)\times Z_{2}^{c}$	1	15
	SO(2)	$Z_{n}\times Z_{2}^{c}$	$S^{1}\;\left[1\leq n\leq 6\right]$	
	I	$I\times Z_{2}^{c}$	1	
	O	$O\times Z_{2}^{c}$	1	
	O	$T\times Z_{2}^{c}$	$Z_{2}$	
	T	$D_{2}\times Z_{2}^{c}$	$Z_{3}$	
	$D_{12}$	$D_{6}\times Z_{2}^{c}$	$Z_{2}$	
	$D_{10}$	$D_{5}\times Z_{2}^{c}$	$Z_{2}$	
	$D_{8}$	$D_{4}\times Z_{2}^{c}$	$Z_{2}$	
	$D_{6}$	$D_{3}\times Z_{2}^{c}$	$Z_{2}$	

**Table 2:** The C-axial subgroups of $O\left(3\right)\times S^{1}$ for the natural representations on $V_{4}⊕V_{4}$. Here $H=J\times Z_{2}^{c}$.

Σ	J	K	θ(H)	Fix(Σ)
$\overset{˜}{O\left(2\right)}$	O(2)	$O\left(2\right)\times Z_{2}^{c}$	1	{(0,0,0,0,z,0,0,0,0)}
$\overset{˜}{O}$	O	$O\times Z_{2}^{c}$	1	$\left\{\left(\sqrt{5}z,0,0,0,\sqrt{14}z,0,0,0,\sqrt{5}z\right)\right\}$
$\overset{˜}{T}$	T	$D_{2}\times Z_{2}^{c}$	$Z_{3}$	$\left\{\left(\sqrt{7}z,0,\sqrt{12}iz,0,-\sqrt{10}z,0,\sqrt{12}iz,0,\sqrt{7}z\right)\right\}$
$\overset{˜}{D_{8}}$	$D_{8}$	$D_{4}\times Z_{2}^{c}$	$Z_{2}$	{(z,0,0,0,0,0,0,0,z)}
$\overset{˜}{D_{6}}$	$D_{6}$	$D_{3}\times Z_{2}^{c}$	$Z_{2}$	{(0,z,0,0,0,0,0,z,0)}
$\overset{˜}{D_{4}}$	$D_{4}$	$D_{2}\times Z_{2}^{c}$	$Z_{2}$	{(0,0,z,0,0,0,z,0,0)}
$\overset{˜}{\mathrm{SO}{\left(2\right)}_{4}}$	SO(2)	$Z_{4}\times Z_{2}^{c}$	$S^{1}$	{(z,0,0,0,0,0,0,0,0)}
$\overset{˜}{\mathrm{SO}{\left(2\right)}_{3}}$	SO(2)	$Z_{3}\times Z_{2}^{c}$	$S^{1}$	{(0,z,0,0,0,0,0,0,0)}
$\overset{˜}{\mathrm{SO}{\left(2\right)}_{2}}$	SO(2)	$Z_{2}\times Z_{2}^{c}$	$S^{1}$	{(0,0,z,0,0,0,0,0,0)}
$\overset{˜}{\mathrm{SO}{\left(2\right)}_{1}}$	SO(2)	$Z_{2}^{c}$	$S^{1}$	{(0,0,0,z,0,0,0,0,0)}

**Table 3:** Isotropy types for possible solutions of (39) together with their representation in the reduced system (42).

	Σ	H	K	Fix(Σ)	${\left|w\right|}^{2}$	${\left|z_{0}^{2}-2z_{-1}z_{1}\right|}^{2}$	${\left|z\right|}^{2}$
1	$\overset{˜}{O\left(3\right)}$	O(3)	O(3)	(w;0,0,0)	> 0	0	0
2	$\overset{˜}{SO\left(2\right)}$	$SO\left(2\right)\times Z_{2}^{c}$	$Z_{2}^{-}$	$\left(0;z_{-1},0,0\right)$	0	0	> 0
3	$\overset{˜}{O\left(2\right)}$	$O\left(2\right)\times Z_{2}^{c}$	$O{\left(2\right)}^{-}$	$\left(0;0,z_{0},0\right)$	0	> 0	> 0
4	$\overset{˜}{Z_{2}}$	$Z_{2}\times Z_{2}^{c}$	$Z_{2}^{-}$	$\left(0;z_{-1},0,z_{1}\right)$	0	> 0	> 0
5	$\overset{˜}{1}$	$Z_{2}^{c}$	1	$\left(0;z_{-1},z_{0},z_{1}\right)$	0	≥ 0	> 0
6	$O{\left(2\right)}^{-}$	$O{\left(2\right)}^{-}$	$O{\left(2\right)}^{-}$	$\left(w;0,z_{0},0\right)$	> 0	> 0	> 0
7	$Z_{2}^{-}$	$Z_{2}^{-}$	$Z_{2}^{-}$	$\left(w;z_{-1},0,z_{1}\right)$	> 0	> 0	> 0
8	1	1	1	$\left(w;z_{-1},z_{0},z_{1}\right)$	> 0	≥ 0	> 0

## Appendix A Spherical harmonics

The spherical harmonics are represented by the functions:

(A.1) $$Y_{n}^{m}\left(\theta ,\phi \right)={\left(-1\right)}^{m}\sqrt{\frac{2n+1}{4\pi }\frac{\left(n-m\right)!}{\left(n+m\right)!}}P_{n}^{m}\left(\cos\theta \right)e^{im\phi }\text{,}\;-n\leq m\leq n\text{,}$$

where

(A.2) $$P_{n}^{m}\left(x\right)=\frac{{\left(1-x^{2}\right)}^{m/2}}{2^{n}n!}\frac{d^{n+m}}{dx^{n+m}}{\left(x^{2}-1\right)}^{n}\text{,}$$

is the associated Legendre function. The spherical harmonics satisfy

(A.3) $$Y_{n}^{-m}\left(\theta ,\phi \right)={\left(-1\right)}^{m}\bar{Y_{n}^{m}}\left(\theta ,\phi \right)\text{.}$$

They also satisfy the orthogonality condition

(A.4) $$\int_{0}^{2\pi }\int_{0}^{\pi }Y_{n}^{m}\left(\theta ,\phi \right)\bar{Y_{n^{\prime }}^{m^{\prime }}\left(\theta ,\phi \right)}\sin\theta d\theta d\phi =\delta_{n,n^{\prime }}\delta_{m,m^{\prime }}\text{.}$$

## Appendix B Basis coefficients

For given $G\left(r,r^{\prime };\mu \right)=w\left(r\cdot r^{\prime }\right)\exp\left(-\mu \tau \left(r\cdot r^{\prime }\right)\right)$, the expansion into spherical harmonics, see Theorem 2, yields coefficients:

$$G_{n}\left(\mu \right)=2\pi \int_{-1}^{1}\left[J_{1}\exp\left(-\frac{\cos^{-1}s}{\sigma_{1}}\right)+J_{2}\exp\left(-\frac{\cos^{-1}s}{\sigma_{2}}\right)\right]\times \exp\left(-\mu \tau_{0}-\frac{\mu \cos^{-1}s}{c}\right)P_{n}\left(s\right)ds=2\pi e^{-\mu \tau_{0}}\left[J_{1}\int_{-1}^{1}\exp\left(-\left(\frac{1}{\sigma_{1}}+\frac{\mu }{c}\right)\cos^{-1}s\right)P_{n}\left(s\right)ds+J_{2}\int_{-1}^{1}\exp\left(-\left(\frac{1}{\sigma_{2}}+\frac{\mu }{c}\right)\cos^{-1}s\right)P_{n}\left(s\right)ds\right]\text{.}$$

For the evaluation of the integrals, we apply the following result. Theorem 4 *For* a∈C *and* $P_{n}$*the Legendre polynomial of degree* n≥0*, the integral*

$$I_{n}\left(a\right)≔\int_{-1}^{1}\exp\left(a\cos^{-1}s\right)P_{n}\left(s\right)ds$$

*satisfies the recurrence relation:*

(B.1) $$I_{n}\left(a\right)I_{n+1}\left(a\right)=\frac{a^{2}\left(1-e^{2a\pi }\right)}{\left(a^{2}+n^{2}\right)\left(a^{2}+{\left(n+1\right)}^{2}\right)\left(a^{2}+{\left(n+2\right)}^{2}\right)}\text{,}\;I_{0}\left(a\right)=\frac{1+e^{a\pi }}{a^{2}+1}\text{.}$$

*This yields, moreover, the two-term recurrence relation:*

$$I_{n+2}\left(a\right)=I_{n}\left(a\right)\frac{a^{2}+n^{2}}{a^{2}+{\left(n+3\right)}^{2}}\text{.}$$

Proof First, consider for n≥0

(B.2) $$H_{n}\left(a\right)≔\int_{0}^{\pi }e^{at}P_{n}\left(\cos t\right)dt\text{.}$$

We will use mathematical induction to show that $H_{n}$ satisfies the following recurrence relation

(B.3) $$H_{n+1}\left(a\right)H_{n}\left(a\right)=\frac{1-e^{2a\pi }}{a^{n}+{\left(n+1\right)}^{2}}\text{,}\;H_{0}\left(a\right)=\frac{e^{a\pi }-1}{a}\text{.}$$

Starting with a recurrence relation for Legendre polynomials, we find

$$\left(n+1\right)P_{n+1}\left(\cos t\right)+nP_{n-1}\left(\cos t\right)=\left(2n+1\right)\cos t\;P_{n}\left(\cos t\right)\text{,}$$

(B.4) $$\left(n+1\right)H_{n+1}\left(a\right)+nH_{n-1}\left(a\right)=\frac{2n+1}{2}\left(H_{n}\left(a+i\right)+H_{n}\left(a-i\right)\right)\text{,}$$

 where we have expressed cost as a complex exponential. Another identity for Legendre polynomials gives

$$P_{n+1}^{\prime }\left(\cos t\right)-P_{n-1}^{\prime }\left(\cos t\right)=\left(2n+1\right)P_{n}\left(\cos t\right)\text{,}$$

$$\int_{0}^{\pi }e^{at}\sin t\left(P_{n+1}^{\prime }\left(\cos t\right)-P_{n-1}^{\prime }\left(\cos t\right)\right)dt=\left(2n+1\right)\int_{0}^{\pi }e^{at}\sin t\;P_{n}\left(\cos t\right)dt\text{,}$$

(B.5) $$a\left(H_{n+1}\left(a\right)-H_{n-1}\left(a\right)\right)=\frac{2n+1}{2i}\left(H_{n}\left(a+i\right)-H_{n}\left(a-i\right)\right)\text{,}$$

 where the left hand side is obtained via integration by parts and the right hand side via expressing sint as a complex exponential. Adding (B.4) and i times (B.5) yields

(B.6) $$\left(n+1+ia\right)H_{n+1}\left(a\right)+\left(n-ia\right)H_{n-1}\left(a\right)=\frac{2n+1}{2}H_{n}\left(a+i\right)\text{.}$$

This identity will be the starting point for the mathematical induction. Assume that (B.3) holds for all n≤N for some N>0; we will prove that it also holds for n=N+1. Combining (B.3) at n=N and at n=N−1, yields

(B.7) $$H_{N-1}\left(a\right)=H_{N+1}\left(a\right)\frac{a^{2}+{\left(N+1\right)}^{2}}{a^{2}+N^{2}}\text{,}$$

which simplifies (B.6):

(B.8) $$H_{N+1}\left(a\right)=\frac{a-iN}{a-i\left(N+1\right)}H_{N}\left(a+i\right)\text{.}$$

Multiply (B.8) with itself at N and N+1 to obtain

$$H_{N+2}\left(a\right)H_{N+1}\left(a\right)=\frac{\left(a-i\left(N+1\right)\right)\left(a-iN\right)}{\left(a-i\left(N+2\right)\right)\left(a-i\left(N+1\right)\right)}H_{N+1}\left(a+i\right)H_{N}\left(a+i\right)\text{,}$$

and apply (B.3) on the right hand side to find

$$H_{N+2}\left(a\right)H_{N+1}\left(a\right)=\frac{1-e^{2a\pi }}{a^{2}+{\left(N+2\right)}^{2}}\text{,}$$

which proves the recurrence relation for $H_{n}\left(a\right)$. Return to $I_{n}\left(a\right)$. Note that for the substitution s=cost:

$$I_{n}\left(a\right)=\int_{0}^{\pi }e^{at}\sin\left(t\right)\;P_{n}\left(\cos t\right)\;dt=\frac{1}{2i}\left(H_{n}\left(a+i\right)-H_{n}\left(a-i\right)\right)\text{.}$$

Substitute this and (B.7) in (B.4) to obtain

(B.9) $$I_{n}\left(a\right)=-\frac{a}{a^{2}+n^{2}}H_{n+1}\left(a\right)\text{.}$$

Multiply the equation with itself at n and n+1

$$I_{n+1}\left(a\right)I_{n}\left(a\right)=\frac{a^{2}}{\left(a^{2}+n^{2}\right)\left(a^{2}+{\left(n+1\right)}^{2}\right)}H_{n+2}\left(a\right)H_{n+1}\left(a\right)\text{,}$$

and apply (B.3) to obtain (B.1). □

## Appendix C Evaluation of critical normal form coefficients

### C.1 Multilinear operators B and C

In the remainder of Appendix A, Appendix B, Appendix C, we denote the second and third derivatives of the full non-linear system by B and C respectively. We define the multilinear operators $B\in L_{2}\left(X,X^{⊙*}\right)$ and $C\in L_{3}\left(X,X^{⊙*}\right)$ as:

(C.1) $$B\left(\eta_{1},\eta_{2}\right)=\left(f^{\prime \prime }\left(\overset{ˆ}{u}\right)\int_{\Omega }w\left(\cdot ,r^{\prime }\right)\overset{2}{\underset{i=1}{\prod }}\eta_{i}\left(t-\tau \left(\cdot ,r^{\prime }\right),\cdot \right)dr^{\prime },0\right)\text{,}$$

(C.2) $$C\left(\eta_{1},\eta_{2},\eta_{3}\right)=\left(f^{\prime \prime \prime }\left(\overset{ˆ}{u}\right)\int_{\Omega }w\left(\cdot ,r^{\prime }\right)\overset{3}{\underset{i=1}{\prod }}\eta_{i}\left(t-\tau \left(\cdot ,r^{\prime }\right),\cdot \right)dr^{\prime },0\right)\text{.}$$

### C.2 Hopf bifurcation, $n_{c}=0$

In this case, let $\lambda =\pm i\omega_{0}$ be the only purely imaginary eigenvalue of the system, such that

$$1+i\omega_{0}-\kappa G_{0}\left(i\omega \right)=0\text{.}$$

This eigenvalue has multiplicity 1 and the corresponding eigenfunction is $\xi \left(t,r\right)=e^{i\omega_{0}t}Y_{0}^{0}\left(r\right)=e^{i\omega_{0}t}/\sqrt{4\pi }$. This case corresponds with the ordinary Hopf bifurcation formulated in  [29] and, hence, we can apply their result directly.

The quadratic terms in the normal form yield equations

(C.3a) $$\left(2i\omega_{0}-A^{⊙*}\right)jh_{20}=B\left(\xi ,\xi \right)\text{,}$$

(C.3b) $$-A^{⊙*}jh_{11}=B\left(\xi ,\bar{\xi }\right)\text{.}$$

 The right hand side of (C.3a) yields

(C.4) $$\left(b_{20},0\right)≔B\left(\xi ,\xi \right)=\left(\frac{f^{\prime \prime }\left(\overset{ˆ}{u}\right)}{4\pi }\int_{\Omega }w\left(\cdot ,r^{\prime }\right)e^{-2i\omega_{0}\tau \left(\cdot ,r^{\prime }\right)}dr^{\prime },0\right)=\left(\frac{f^{\prime \prime }\left(\overset{ˆ}{u}\right)}{4\pi }\int_{\Omega }G\left(\cdot ,r^{\prime };2i\omega_{0}\right)dr^{\prime },0\right)\text{.}$$

Combining (35a) and Theorem 3 gives

(C.5) $$h_{20}\left(t,r\right)=e^{2i\omega_{0}t}\overset{\infty }{\underset{n=0}{\sum }}\overset{n}{\underset{m=-n}{\sum }}\frac{{\left\{b_{20}\right\}}_{n}^{m}}{2i\omega_{0}+1-\kappa G_{n}\left(2i\omega_{0}\right)}Y_{n}^{m}\left(r\right)\text{,}$$

where ${\left\{b_{20}\right\}}_{n}^{m}$ denotes the coefficients from the spherical harmonic expansion of $b_{20}$; see Theorem 1. Using spherical harmonic expansion and orthonormality, we find

$${\left\{b_{20}\right\}}_{n}^{m}≔\frac{f^{\prime \prime }\left(\overset{ˆ}{u}\right)}{4\pi }\int_{\Omega }\int_{\Omega }G\left(r,r^{\prime };2i\omega_{0}\right)\bar{Y_{n}^{m}\left(r\right)}drdr^{\prime }=\frac{f^{\prime \prime }\left(\overset{ˆ}{u}\right)}{4\pi }\overset{\infty }{\underset{n^{\prime }=0}{\sum }}G_{n^{\prime }}\left(2i\omega_{0}\right)\overset{n^{\prime }}{\underset{m^{\prime }=-n^{\prime }}{\sum }}\int_{\Omega }\int_{\Omega }Y_{n^{\prime }}^{m^{\prime }}\left(r\right)\bar{Y_{n^{\prime }}^{m^{\prime }}\left(r^{\prime }\right)Y_{n}^{m}\left(r\right)}drdr^{\prime }=\frac{f^{\prime \prime }\left(\overset{ˆ}{u}\right)}{4\pi }G_{n}\left(2i\omega_{0}\right)\int_{\Omega }\bar{Y_{n}^{m}\left(r^{\prime }\right)}dr^{\prime }=\left\{ \begin{array}{cc} \frac{f^{\prime \prime }\left(\overset{ˆ}{u}\right)}{2\sqrt{\pi }}G_{0}\left(2i\omega_{0}\right) & n=0\\ 0 & n>0\text{.} \end{array}\right.$$

The last step results from properties of the spherical harmonics. Hence:

$$h_{20}\left(t,r\right)=\frac{f^{\prime \prime }\left(\overset{ˆ}{u}\right)}{2\sqrt{\pi }}Q_{0}\left(2i\omega_{0}\right)e^{2i\omega_{0}t}Y_{0}^{0}\left(r\right)$$

where we have defined

$$Q_{n}\left(\mu \right)≔\frac{G_{n}\left(\mu \right)}{\mu +1-\kappa G_{n}\left(\mu \right)}\text{.}$$

In a similar manner, we solve (C.3b)

$$h_{11}\left(t,r\right)=\frac{f^{\prime \prime }\left(\overset{ˆ}{u}\right)}{2\sqrt{\pi }}Q_{0}\left(0\right)Y_{0}^{0}\left(r\right)\text{.}$$

The cubic terms of the Hopf bifurcation give rise to the equation

(C.6) $$\frac{1}{2}\left(i\omega_{0}-A^{⊙*}\right)jh_{21}=\left(v,0\right)-g_{21}j\xi \text{,}$$

with v such that

(C.7) $$\left(v,0\right)≔\frac{1}{2}C\left(\xi ,\xi ,\bar{\xi }\right)+\frac{1}{2}B\left(\bar{\xi },h_{20}\right)+B\left(\xi ,h_{11}\right)\text{,}$$

because B and C are zero in the second component. We apply (35b) to obtain

$$g_{21}Y_{0}^{0}=\frac{1}{2\pi i}\oint_{\partial D_{i\omega_{0}}}\Delta {\left(\mu \right)}^{-1}v\;d\mu \text{.}$$

Expansion of both sides into spherical harmonics results in an equation for each coefficient

$$\begin{array}{ccc} g_{21} & = & \frac{1}{2\pi i}\oint_{\partial D_{i\omega_{0}}}\frac{v_{0}^{0}}{\mu +1-\kappa G_{0}\left(\mu \right)}d\mu \text{,}\\ 0 & = & \frac{1}{2\pi i}\oint_{\partial D_{i\omega_{0}}}\frac{v_{n}^{m}}{\mu +1-\kappa G_{n}\left(\mu \right)}d\mu \;n>0,m\leq \left|n\right|\text{.} \end{array}$$

From the characteristic equation it follows that $i\omega_{0}+1-\kappa G_{0}\left(i\omega_{0}\right)=0$, and we also have that $\mu +1-\kappa G_{n}\left(\mu \right)\neq 0$ for all n>0. Therefore, in the second equation the integrand is analytic for all n>0 and these contour integrals vanish. The first equation is evaluated using Cauchy’s integral formula:

$$g_{21}=\frac{v_{0}^{0}}{1-\kappa G_{0}^{\prime }\left(i\omega \right)}\text{,}$$

where $G_{0}^{\prime }$ denotes the derivative of $G_{0}$. Next, (C.7) yields

(C.8) $$v\left(r\right)=\frac{1}{16\pi^{3/2}}\left(f^{\prime \prime \prime }\left(\overset{ˆ}{u}\right)+Q_{0}\left(2i\omega_{0}\right)f^{\prime \prime }{\left(\overset{ˆ}{u}\right)}^{2}+2Q_{0}\left(0\right)f^{\prime \prime }{\left(\overset{ˆ}{u}\right)}^{2}\right)\int_{\Omega }G\left(r,r^{\prime };i\omega_{0}\right)dr^{\prime }\text{,}$$

and hence

$$v_{n}^{m}=\left\{ \begin{array}{c} \frac{1}{8\pi }G_{0}\left(i\omega_{0}\right)\left(f^{\prime \prime \prime }\left(\overset{ˆ}{u}\right)+f^{\prime \prime }{\left(\overset{ˆ}{u}\right)}^{2}\left[Q_{0}\left(2i\omega_{0}\right)+2Q_{0}\left(0\right)\right]\right)n=0\\ 0\;n>0\text{.} \end{array}\right.$$

Therefore, we find the critical normal form coefficient to be

(C.9) $$g_{21}=\frac{1+i\omega_{0}}{8\pi \kappa \left(1-\kappa G_{0}^{\prime }\left(i\omega_{0}\right)\right)}\left(f^{\prime \prime \prime }\left(\overset{ˆ}{u}\right)+f^{\prime \prime }{\left(\overset{ˆ}{u}\right)}^{2}\left[Q_{0}\left(2i\omega_{0}\right)+2Q_{0}\left(0\right)\right]\right)\text{,}$$

where we used the result from the characteristic equation that $G_{0}\left(i\omega_{0}\right)=\left(1+i\omega_{0}\right)/\kappa$.

### C.3 Hopf bifurcation, $n_{c}=1$

For n=1, there is one pair of purely imaginary eigenvalues $\lambda =\pm i\omega_{1}$ such that

(C.10) $$1+i\omega_{1}-\kappa G_{1}\left(i\omega_{1}\right)=0\text{.}$$

In this case, the eigenvalue has algebraic and geometric multiplicity 3, with corresponding eigenfunctions $\xi_{m}\left(t,r\right)=e^{i\omega_{1}t}Y_{1}^{m}\left(r\right)$ for m=−1,0,1. The corresponding Poincaré normal form, to which the system is smoothly orbitally equivalent, is of the form:

(C.11) $${\overset{˙}{z}}_{m}=i\omega_{1}z_{m}+g_{\left(3,1\right)}z_{m}{\left|z\right|}^{2}+g_{\left(3,2\right)}{\overset{ˆ}{z}}_{m}\left(z_{0}^{2}-2z_{-1}z_{1}\right)+O\left({\left|z\right|}^{4}\right)\text{,}\;m=-1,0,1\text{,}$$

$$z≔\left(z_{-1},z_{0},z_{1}\right)\text{,}\;\overset{ˆ}{z}≔\left(-\bar{z_{1}},\bar{z_{0}},-\bar{z_{-1}}\right)\text{,}\;{\left|z\right|}^{2}≔\overset{1}{\underset{m=-1}{\sum }}{\left|z_{m}\right|}^{2}\text{.}$$

 with $g_{\left(3,1\right)}$ and $g_{\left(3,2\right)}$ the critical normal form coefficients belonging to the two equivariants of order 3, see for instance  [63]. The critical centre manifold is now of the form

(C.12) $$H\left(z,\bar{z}\right)=\overset{1}{\underset{m=-1}{\sum }}z_{m}\xi_{m}+\bar{z_{m}\xi_{m}}+\underset{\left|\nu \right|+\left|\zeta \right|\geq 2}{\sum }\frac{1}{\nu !\zeta !}h_{\nu \;\zeta }\;z^{\nu }{\bar{z}}^{\zeta }\text{,}$$

where ν and ζ are multi-indices of length 3, such that

$$\left|\nu \right|≔\overset{1}{\underset{m=-1}{\sum }}\left|\nu_{m}\right|\text{,}\;\nu !≔\overset{1}{\underset{m=-1}{\prod }}\nu_{m}!\text{,}\;z^{\nu }≔\overset{1}{\underset{m=-1}{\prod }}z_{m}^{\nu_{m}}\text{.}$$

After substituting (C.11), (C.12) into (34) we obtain one large equation that we solve by equating powers of z and $\bar{z}$ on both sides. At second order, we are able to find an equation for each of the 21 $h_{\nu \;\zeta }$, but here we list only those that we will require to determine the critical coefficients g:

(C.13a) $$\left(2i\omega_{1}-A^{⊙*}\right)jh_{200\;000}=B\left(\xi_{-1},\xi_{-1}\right)\text{,}$$

(C.13b) $$-A^{⊙*}jh_{100\;100}=B\left(\xi_{-1},\bar{\xi_{-1}}\right)\text{,}$$

(C.13c) $$\left(2i\omega_{1}-A^{⊙*}\right)jh_{020\;000}=B\left(\xi_{0},\xi_{0}\right)\text{,}$$

(C.13d) $$-A^{⊙*}jh_{010\;001}=B\left(\xi_{0},\bar{\xi_{1}}\right)\text{.}$$

 Note that each of these equations is of the form (C.3) and is solved in an identical manner: evaluate B, expand in spherical harmonics and apply the resolvent. In particular, one will encounter integrals involving the product of three spherical harmonics for which the following identity is useful:

$$\int_{\Omega }Y_{n_{1}}^{m_{1}}\left(r\right)Y_{n_{2}}^{m_{2}}\left(r\right)Y_{n_{3}}^{m_{3}}\left(r\right)\;dr=\sqrt{\frac{\left(2n_{1}+1\right)\left(2n_{2}+1\right)\left(2n_{3}+1\right)}{4\pi }}\left(\begin{array}{ccc} n_{1} & n_{2} & n_{3}\\ 0 & 0 & 0 \end{array}\right)\left(\begin{array}{ccc} n_{1} & n_{2} & n_{3}\\ m_{1} & m_{2} & m_{2} \end{array}\right)\text{,}$$

where the terms in parentheses are the Wigner 3j symbols  [64, Section 34]. For example, we find for $h_{200\;000}$

$$B\left(\xi_{-1},\xi_{-1}\right)=\left(\sqrt{\frac{3}{10\pi }}f^{\prime \prime }\left(\overset{ˆ}{u}\right)G_{2}\left(2i\omega_{1}\right)Y_{2}^{-2},0\right)\text{,}$$

$$h_{200\;000}\left(t,r\right)=\sqrt{\frac{3}{10\pi }}f^{\prime \prime }\left(\overset{ˆ}{u}\right)Q_{2}\left(2i\omega_{1}\right)\;e^{2i\omega_{1}t}\;Y_{2}^{-2}\left(r\right)\text{,}$$

 and similar expressions for the remaining three h’s.

At cubic order, the homological equation yields the following two equations (out of a total of 56)

$$\left(i\omega_{1}-A^{⊙*}\right)jh_{200\;100}=\left(v_{1},0\right)+2g_{\left(3,1\right)}j\xi_{-1}\text{,}$$

$$\left(i\omega_{1}-A^{⊙*}\right)jh_{020\;001}=\left(v_{2},0\right)-2g_{\left(3,2\right)}j\xi_{-1}\text{,}$$

 where

$$\left(v_{1},0\right)≔C\left(\xi_{-1},\xi_{-1},\bar{\xi_{-1}}\right)+B\left(\bar{\xi_{-1}},h_{200\;000}\right)+2B\left(\xi_{-1},h_{100\;100}\right)\text{,}$$

$$\left(v_{2},0\right)≔C\left(\xi_{0},\xi_{0},\bar{\xi_{1}}\right)+B\left(\bar{\xi_{1}},h_{020\;000}\right)+2B\left(\xi_{0},h_{010\;010}\right)\text{.}$$

 Note the similarity of these equations with (C.6), (C.7). Hence, using the same procedure as before, we find:

(C.14a) $$g_{\left(3,1\right)}=\frac{1+i\omega_{1}}{20\pi \kappa \left(1-\kappa G_{1}^{\prime }\left(i\omega_{1}\right)\right)}\left(3f^{\prime \prime \prime }\left(\overset{ˆ}{u}\right)+f^{\prime \prime }{\left(\overset{ˆ}{u}\right)}^{2}\left[5Q_{0}\left(0\right)+Q_{2}\left(0\right)+3Q_{2}\left(2i\omega_{1}\right)\right]\right)\text{,}$$

(C.14b) $$g_{\left(3,2\right)}=\frac{1+i\omega_{1}}{40\pi \kappa \left(1-\kappa G_{1}^{\prime }\left(i\omega_{1}\right)\right)}\left(3f^{\prime \prime \prime }\left(\overset{ˆ}{u}\right)+f^{\prime \prime }{\left(\overset{ˆ}{u}\right)}^{2}\left[5Q_{0}\left(0\right)+6Q_{2}\left(0\right)-2Q_{2}\left(2i\omega_{1}\right)\right]\right)\text{.}$$

 An important remark is that we could have selected a different set of equations to determine the coefficients, yet their outcome will be identical due to the symmetries in the system.

### C.4 Double Hopf bifurcation, $n_{c}=\left\{0,1\right\}$

At the Hopf–Hopf bifurcation, the system has two pairs of purely complex eigenvalues, namely $\lambda =\pm i\omega_{0}$ (with multiplicity 1) and $\lambda =\pm i\omega_{1}$ (with multiplicity 3). The corresponding eigenfunctions are $\psi \left(t,r\right)=e^{i\omega_{0}t}Y_{0}^{0}\left(r\right)$ and $\xi_{m}\left(t,r\right)=e^{i\omega_{1}t}Y_{1}^{m}\left(r\right)$ for m∈{−1,0,1}. In the absence of resonances, the Poincaré normal form is the following (truncated at cubic order):

$$\overset{˙}{w}=i\omega_{0}w+g_{21}w\left|w^{2}\right|+\gamma_{0}w{\left|z\right|}^{2}+O\left({\left|z\right|}^{4}\right)\text{,}$$

$${\overset{˙}{z}}_{m}=i\omega_{1}z_{m}+g_{\left(3,1\right)}z_{m}{\left|z\right|}^{2}+g_{\left(3,2\right)}{\overset{ˆ}{z}}_{m}\left(z_{0}^{2}-2z_{-1}z_{1}\right)+\gamma_{1}z_{m}{\left|w\right|}^{2}+O\left({\left|z\right|}^{4}\right)\text{,}\;m=-1,0,1\text{,}$$

 where $z≔\left(w,z_{-1},z_{0},z_{1}\right)$ and |z| as in the previous section. Note how the equations for w and $z_{m}$ correspond with the normal forms in the Hopf bifurcations, (C.11), (36) respectively, with the exception of the mixed terms. Hence, the critical normal form coefficients $g_{21}$, $g_{\left(3,1\right)}$ and $g_{\left(3,2\right)}$ are given by (C.9), (C.9) and (C.14). We are left to compute the coefficients $\gamma_{0}$ and $\gamma_{1}$.

Following the same procedure as in Appendix C.3, we construct the homological equation. At quadratic order, we obtain:

(C.15a) $$\left(i\omega_{0}+i\omega_{1}-A^{⊙*}\right)jh_{1010\;0000}=B\left(\psi ,\xi_{0}\right)\text{,}$$

(C.15b) $$\left(i\omega_{0}-i\omega_{1}-A^{⊙*}\right)jh_{1000\;0010}=B\left(\psi ,\bar{\xi_{0}}\right)\text{,}$$

(C.15c) $$-A^{⊙*}jh_{0010\;0010}=B\left(\xi_{0},\bar{\xi_{0}}\right)\text{,}$$

(C.15d) $$\left(i\omega_{0}+i\omega_{1}-A^{⊙*}\right)jh_{1100\;0000}=B\left(\psi ,\xi_{-1}\right)\text{,}$$

(C.15e) $$\left(i\omega_{0}-i\omega_{1}-A^{⊙*}\right)jh_{1000\;0010}=B\left(\psi ,\bar{\xi_{-1}}\right)\text{,}$$

(C.15f) $$\left(-i\omega_{0}+i\omega_{1}-A^{⊙*}\right)jh_{0100\;1000}=B\left(\xi_{-1},\bar{\psi }\right)\text{,}$$

 which can all be solved to obtain $h_{\nu \;\zeta }$. At cubic order, we have the following non-singular equations:

(C.16a) $$\left(i\omega_{0}-A^{⊙*}\right)jh_{1010\;0010}=\left(v_{0},0\right)-\gamma_{0}j\psi \text{,}$$

(C.16b) $$\left(i\omega_{1}-A^{⊙*}\right)jh_{1100\;1000}=\left(v_{1},0\right)-\gamma_{1}j\xi_{-1}\text{,}$$

 where

(C.17a) $$\left(v_{0},0\right)≔C\left(\psi ,\xi_{0},\bar{\xi_{0}}\right)+B\left(\psi ,h_{0010\;0010}\right)+B\left(\xi_{0},h_{1000\;0010}\right)+B\left(\bar{\xi_{0}},h_{1010\;0000}\right)\text{,}$$

(C.17b) $$\left(v_{1},0\right)≔C\left(\psi ,\xi_{-1},\bar{\psi }\right)+B\left(\psi ,h_{0100\;1000}\right)+B\left(\xi_{-1},h_{1000\;0010}\right)+B\left(\bar{\xi_{-1}},h_{1100\;0000}\right)\text{.}$$

 From the solvability conditions, we find expressions for the critical normal form coefficients:

$$\gamma_{0}=\frac{1+i\omega_{0}}{4\pi \kappa \left(1-\kappa G_{0}^{\prime }\left(i\omega_{0}\right)\right)}\left(f^{\prime \prime \prime }\left(\overset{ˆ}{u}\right)+f^{\prime \prime }{\left(\overset{ˆ}{u}\right)}^{2}\left[Q_{0}\left(0\right)+Q_{1}\left(i\omega_{0}+i\omega_{1}\right)+Q_{1}\left(i\omega_{0}-i\omega_{1}\right)\right]\right)\text{,}$$

$$\gamma_{1}=\frac{1+i\omega_{1}}{4\pi \kappa \left(1-\kappa G_{1}^{\prime }\left(i\omega_{1}\right)\right)}\left(f^{\prime \prime \prime }\left(\overset{ˆ}{u}\right)+f^{\prime \prime }{\left(\overset{ˆ}{u}\right)}^{2}\left[Q_{0}\left(0\right)+Q_{1}\left(i\omega_{0}+i\omega_{1}\right)+Q_{1}\left(-i\omega_{0}+i\omega_{1}\right)\right]\right)\text{.}$$

## Footnotes

1 For ease of notation, we will sometimes use η(t)(r) instead of η(t,r).
